# Antiradical and Antioxidant Activity of Compounds Containing 1,3-Dicarbonyl Moiety: An Overview

**DOI:** 10.3390/molecules28176203

**Published:** 2023-08-23

**Authors:** Laima Bērziņa, Inese Mieriņa

**Affiliations:** Institute of Technology of Organic Chemistry, Faculty of Materials Science and Applied Chemistry, Riga Technical University, LV-1048 Riga, Latvia; laima.berzina@inbox.lv

**Keywords:** antiradical activity, antioxidant activity, 1,3-dicarbonyl compound, β-dicarbonyl compound, 1,3-diketone, keto–enol tautomerism

## Abstract

Free radicals and oxidants may cause various damages both to the lifeworld and different products. A typical solution for the prophylaxis of oxidation-caused conditions is the usage of various antioxidants. Among them, various classes are found—polyphenols, conjugated polyalkenes, and some sulfur and nitrogen derivatives. Regarding the active site in the molecules, a widely discussed group of compounds are 1,3-dicarbonyl compounds. Among them are natural (e.g., curcumin and pulvinic acids) and synthetic (e.g., 4-hydroxy coumarins, substituted Meldrum’s acids) compounds. Herein, information about various compounds containing the 1,3-dicarbonyl moiety is covered, and their antiradical and antioxidant activity, depending on the structure, is discussed.

## 1. Introduction

Free radicals and oxidants are an integral part of an aerobic world like planet Earth. Our bodies naturally produce reactive oxygen and nitrogen species in various endogenous systems [1]. However, their roles are like two sides of a coin—they may be helpful and harmful to our bodies [2]. Reactive oxygen species are involved in various physiological processes as signal mediators [3]. On the other hand, free radicals cause damage to organs, cells, mitochondria, and bio-molecules. These processes result in oxidative stress, and aging occurs [4]. Oxidative stress may cause various chronic and degenerative illnesses [2], e.g., cardiovascular conditions [2,3,5], cancer, autoimmune disorders [2], etc. Furthermore, oxidized lipids, proteins, and nucleic acids have been detected in the brain tissue of people who have Parkinson’s disease [6]. Overproduction of reactive oxygen species (ROS) alternates the mammalian target of rapamycin (mTOR) pathway, which is associated with the development of Alzheimer’s disease [7]. Oxidative stress has demonstrated a dual role in cancer cells: the formation of ROS stimulates cellular damage and mutations, thus promoting the development of a tumor; simultaneously, the overexposure of cancer cells to ROS may cause their apoptosis [8].

**Figure 1:**
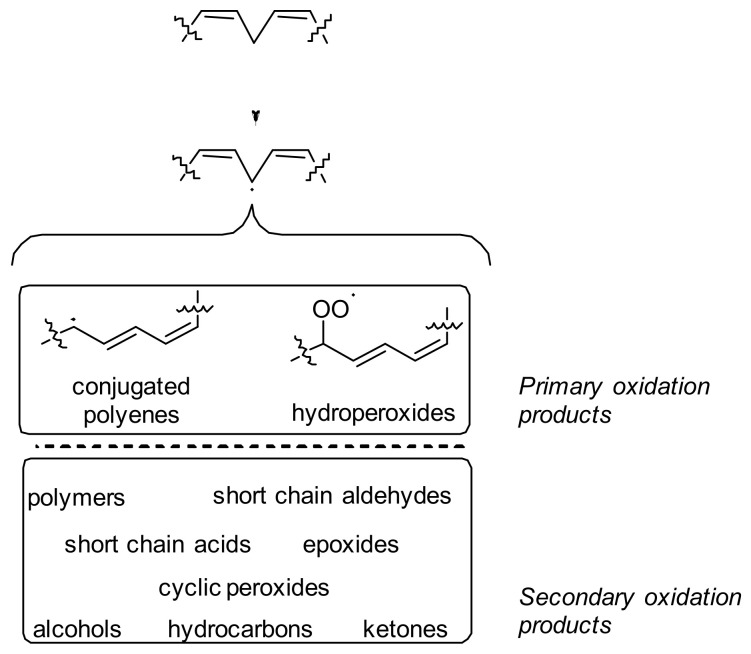
Autooxidation of polyunsaturated lipids. Autooxidation is initiated in the position with the lowest bond dissociation energy—usually the autooxidation in fatty acid triglycerides starts at the *bis*-allyl position, followed by the allyl position. The generated carbon radical is suspected to rearrange, leading to an allyl-type radical. A later reaction with oxygen leads to a peroxyl radical. The isomerization, scission, rearrangement, and polymerization of primary oxidation products generates secondary oxidation products.

In addition to affecting complex processes in the human body, it is well established that various products, from food and medicine to technical applications, containing (poly)unsaturated fatty acid derivatives are suspected of autooxidation. Some recent reviews have covered lipid peroxidation kinetic studies [9], lipid oxidation in emulsions [10], autooxidation of both triglycerides and minor components in extra-virgin olive oil during cooking [11], and the oxidative stability of biodiesel [12] issues. Briefly, lipid autooxidation is a free radical reaction cascade involving initiation (the formation of lipid radicals) and propagation, leading to various peroxides. Then, these peroxides, via cyclization, rearrangement, scission, and condensation, terminate the reaction chain, leading to secondary oxidation products (Figure 1). Heat- [13] and sunlight- [14] induced collapse of various polymer materials results from rapid autoxidation. In contrast, oxidation-response polymers have found application in medicine [15]. The oxidation of polyunsaturated fats in food products results in oxidative rancidity [16]. Typically, for minimizing autooxidation processes and increasing the shelf-life, various antioxidants are added before the oxidation process develops uncontrollably. Polyphenols are well-known tools for controlling such undesired changes in the molecular structure of foods [17]. It is worth mentioning that free radicals have turned into valuable soldiers for the degradation of microplastics [18] and wastewater treatment [19,20].

Different antioxidants may act as influential players in the prophylaxis of disorders caused by oxidative stress. Anthocyanins have been reported to be protective against atherosclerosis and cardiovascular disease [21]. Dietary flavonoids have demonstrated inhibition of low-density lipoprotein (LDL) oxidation and platelet aggregation. Khan et al. have even speculated that moderate consumption of red wine, due to its flavonoids, may reduce the risk of atherosclerosis and thrombosis [22].

Several mechanisms have been provided for explaining the reactions between free radicals and antioxidants. The role of phenol-type antioxidants in hindering the Fenton reaction by complexing both Fe^3+^ and Fe^2+^ ions is well established [23]. On the other hand, carnosic acid effectively neutralizes peroxyl radicals, leading to quinoid structures [24]. Experimental and theoretical studies have demonstrated that disulfides scavenge HO^•^ via cleavage of the -S-S- bond [25]. The mechanism strongly depends on the media; e.g., it is postulated that phenylpropanoids react with free radicals via electron transfer and sequential proton transfer in solvents with a low ionizing ability [26]. It is worth mentioning that it is not always possible to distinguish which mechanism is preferable for explaining antioxidant and/or antiradical activity. E.g., potentiometric studies of various flavonoids and coumarins have highlighted that their activity is due to synergism among electron transfer, hydrogen atom transfer, and metal chelation mechanisms [27]. The density functional theory calculations for various ingredients of essential oils have turned to the hydrogen atom transfer (HAT) mechanism as the most favorable in nonpolar solvents, whilst the sequential proton loss and electron transfer (SPLET) mechanism is dominant in polar solutions [28]. If the antioxidant contains only one structural moiety responsible for antiradical or antioxidant activity, the deduction of the most plausible mechanism is relatively easy. The situation is complex when the structure contains several units, which may affect the activity. E.g., the natural polyphenol silybin contains several hydroxy groups, and each of them has a different role in providing antiradical and/or antioxidant activity [29]. When new antioxidants are constructed, it is usually considered if the antioxidant can scavenge several free radicals: e.g., coumarin–chalcone hybrids are effective for two SPLET and two HAT cycles [30].

Recent studies on antioxidants, mainly covering antioxidative vitamins, polyphenols, and enzymatic antioxidants, have been reviewed by Parcheta et al. [31]. Various natural antioxidants are established as useful for preventing UV radiation-induced ROS, thus turning them into promising agents for cosmetical applications, for example, in sunscreens [32]. A few classes of common antioxidants—carotenoids [33,34], different polyphenols (flavonoids [34,35], chalcones [36], and anthocyanins [34,37]), and coumarins [38,39]—have been well-reviewed in the last several years. Another structurally differential class of compounds—spiro-cyclic systems [40]—have been highlighted as potential antioxidants and free radical scavengers. Nano-science has revealed various nanoparticles, like CeO_2_ [41], nano-selenium, nano-zinc [42], and nitroxides [43], as promising antioxidative scaffolds. Nanostructured forms of organic materials are not an exception: e.g., lignin seems to be a promising antioxidant and UV shield in sunscreen [44]. Polymeric materials have turned into a promising solution for delivering antioxidants [45].

To date, there has been no review covering various antioxidants containing the 1,3-dicarbonyl moiety. Thus, this review covers another exciting class of antioxidants—CH acids containing *β*-dicarbonyl moieties. The structure–activity relationship is analyzed, and the role of dicarbonyl moiety is discussed.

One of the most widely explored 1,3-dicarbonyl compounds is curcumin. It naturally occurs (up to 2%) mainly in turmeric (*Curcuma longa*) [46]. Since ancient times, the benefits of phytochemical curcumin have been applied in traditional medicine [47]. In recent years, several reviews highlighting various biological activities of curcumin have seen the light of day, indicating that curcumin is a green anticancer weapon [48,49,50,51,52,53,54]. The wide application of curcumin in cancer therapy has forced Kong et al. [55] to ask whether “curcumin is the answer to future chemotherapy cocktail?”. This compound has been found to be helpful for the treatment of various diseases caused by free radicals: e.g., metabolic-associated fatty liver disease [56], Huntington’s disease [57], diabetes mellitus [58] and diabetic complications [59], neurodegenerative diseases [60,61], and dermatological disorders [62]. In addition, both structural modifications of curcumin [49,63] and the synthesis of metal complexes [64] have led to compounds with promising pharmacological effects. It should be noted that the outstanding properties of curcumin have encouraged the development of nanoformulations for cancer treatment [65,66], therapy for aging-related diseases [67], and antimicrobial purposes [68]. Guo et al. analyzed curcumin’s plausible antioxidant activity mechanism [69]. However, no attention has been turned to structure–activity analysis. Hunyadi covered the metabolic processes of curcumin in the body [70]. The special biological activities of curcumin have encouraged effective isolation and purification [71,72,73,74], conventional synthetic [74], and biosynthetic [75] routes.

## 2. An Overview of the Method Used for Testing Antioxidant and Antiradical Activity

A general overview of the various methods available for testing antioxidant and antiradical activity was published by Carlos et al. [76]. Briefly, two types of methods are applied: (1) reactions between antioxidant molecules and the free radicals, and (2) processes where a real system is suspected to rapidly oxidize and the decrease in the oxidation of the substrate in the presence and absence of the antioxidant is monitored. The term “antiradical activity” should be attributed to the methods where the test compound scavenges the free radicals. The term “antioxidant activity” is provided for methods where the test compound is used for inhibiting the oxidation of the substrate [77]. The main advantages of the first group are as follows: usually, the reactions are rather fast, standard laboratory equipment (UV-Vis spectrometer) is required, the solubility of the antioxidants can be achieved, and the reaction products may be isolated and characterized. On the other hand, often, the used radicals are not naturally occurring, and the results may not correlate with the activity in real systems. The main advantages of the second group of methods are the following: the antioxidant is tested in the presence of an oxidative unstable substrate and the obtained results may be characteristic for predicting antioxidant effectivity in similar real products. Some of the drawbacks of these methods may include rather long experiment times, depending on the substrate solubility limitations of plausible antioxidants, and in some cases, rather specific equipment may be required.

### 2.1. Tests for Determining Antiradical Activity

Widely used antiradical assays involve the reaction with 2,2-diphenyl-1-picrylhydrazyl (DPPH), galvinoxyl (GO), 2,2′-azino-*bis*(3-ethylbenzothiazoline-6-sulfonic acid) radical cation (ABTS), and hydroxyl radical. DPPH and GO are commercial stable free radicals. In cases of other test systems, the free radical or radical cation should be generated prior to the experiment.

**Scheme 1:**
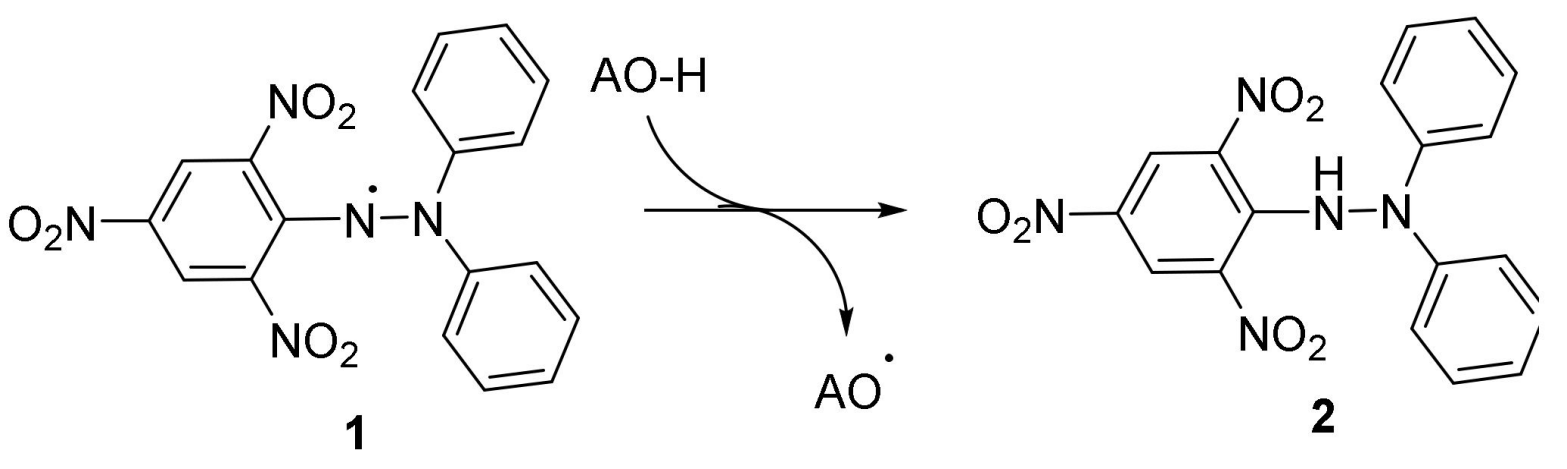
Representative reaction between DPPH and an antioxidant.

The DPPH test (Scheme 1) is widely used to evaluate the antiradical activity of both individual compounds and plant extracts. Typically, it is a rapid, spectrophotometric method used for solutions. An overview of this assay was published by Kedare et al. [78]. The quenching of DPPH **1** is monitored at λ = 515 nm. The reaction is run in any solvent; however, widely used solvents are ethanol and methanol. The reaction rate strongly depends on the solvents; it was established that the reaction is faster in polar solvents than in unpolar ones [79]. Some modern DPPH assays involve electrochemical principles [80], chromatographic routes like thin layer chromatography (TLC) on silica plates [81] or high-performance liquid chromatography (HPLC) [82], as well as the application of analyte (solid sample [83] or the sample doped on silica and modified silica plates [84]) or DPPH (immobilized in 96-well plates [85], pharmaceutical blisters [86], or paper [87]) in solid form.

**Scheme 2:**
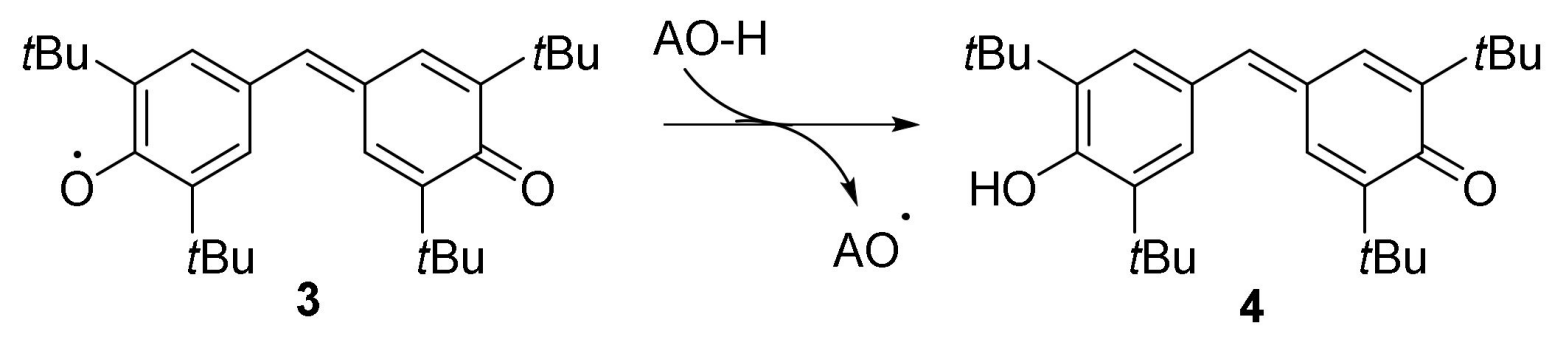
Representative reaction between GO and an antioxidant.

A less used free radical for the rapid testing of antiradical activity is galvinoxyl **3** (GO) (Scheme 2). Similarly to DPPH assays, the test is implemented in a solution, and the absorbance (λ = 428 nm [88]) after a certain time is registered [89]. Other routes for monitoring GO inhibition efficiency involve electron spin resonance [90], HPLC with a post-column injection of GO [91], and electrochemical assay with galvinoxyl immobilized on electrodes [92].

**Scheme 3:**
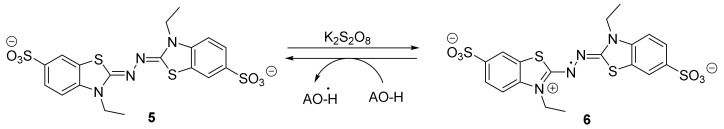
Generation of ABTS radical cation and its reaction with an antioxidant.

Contrary to the DPPH and GO tests, ABTS radical cation **6** (Scheme 3) should be generated from the corresponding acid **5** prior to the analysis. A typical reagent for this transformation is potassium persulfate [93], but faster electrolysis may be useful too [94]. The amount of the radical cation is monitored by a spectrophotometer (λ = 730…750 nm) [93]. Similarly to GO and DPPH, an HPLC post-column treatment with a radical cation is elaborated for ABTS assay [95].

**Scheme 4:**
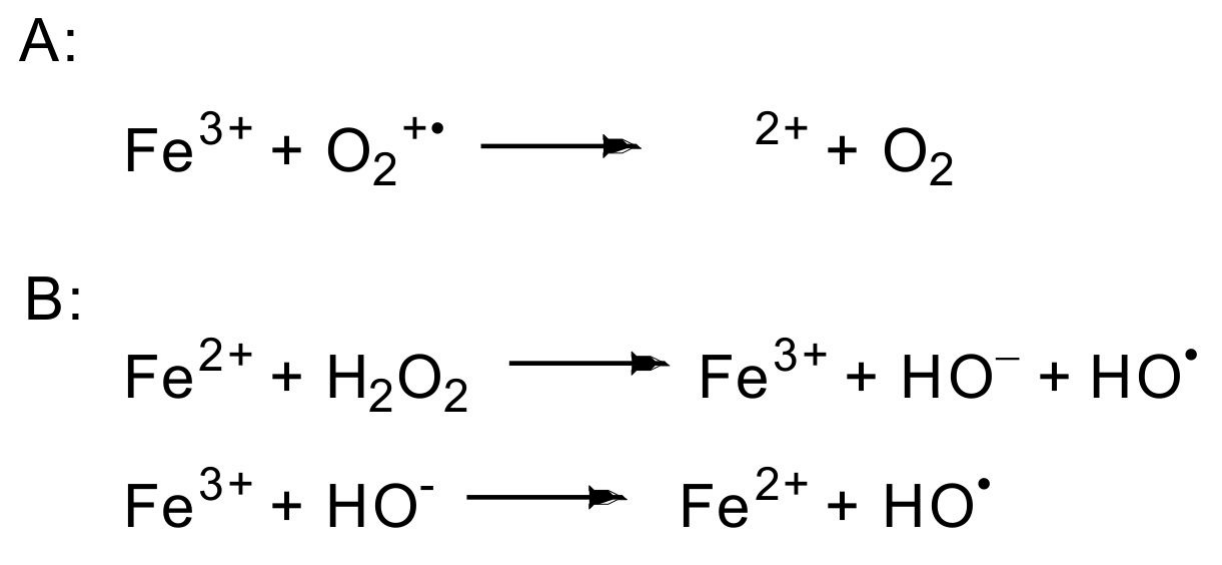
Fe^2+^ ions are produced in the Haber–Weiss reaction (**A**), and then, in their reaction with hydrogen peroxide, hydroxyl radicals are formed (**B**). Hydroxyl radical formation from hydroxide ions may be catalyzed by Fe^3+^.

**Scheme 5:**
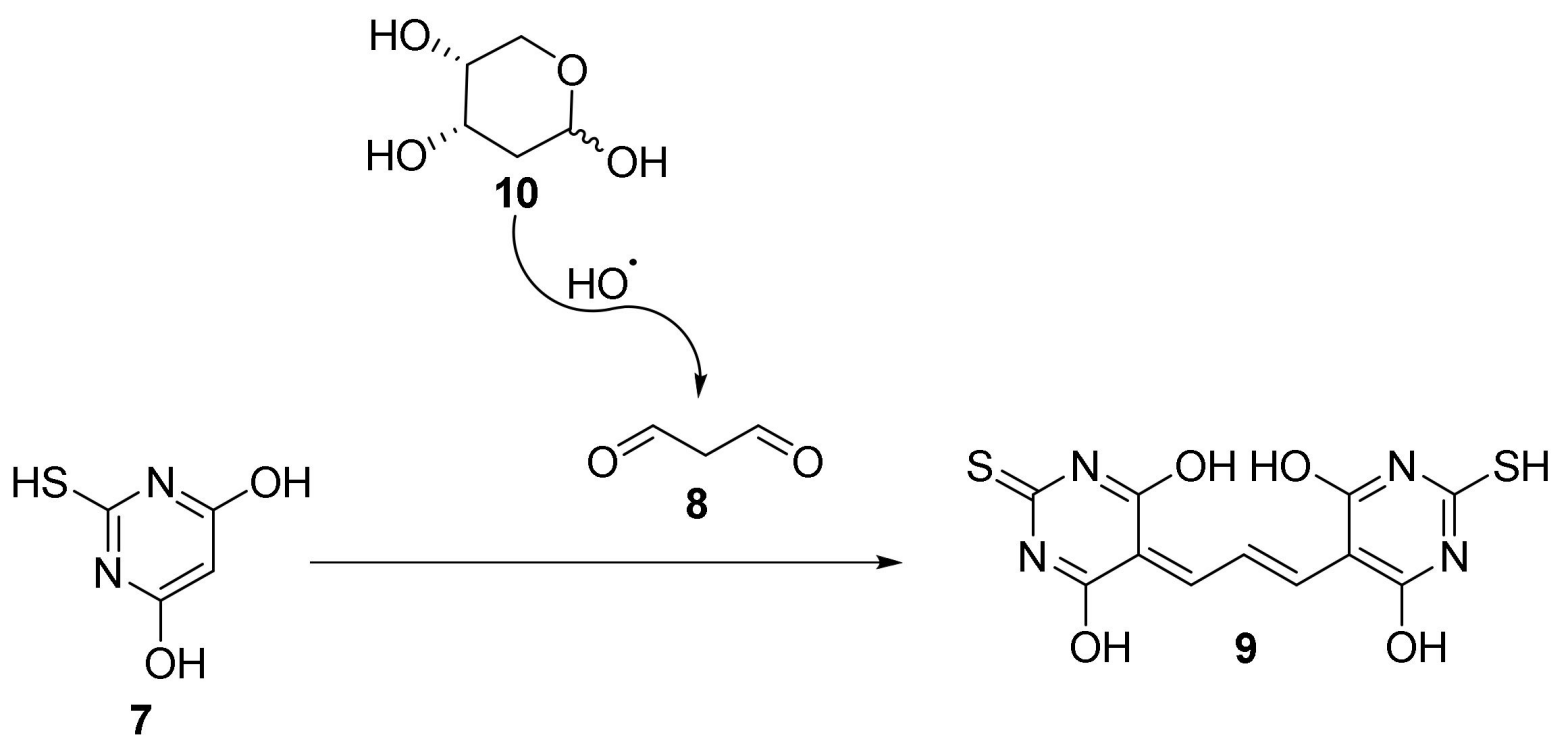
The principle for testing antioxidants for their ability to scavenge hydroxyl radicals: the hydroxyl radicals convert 2-deoxy-d-ribose into malonaldehyde. The latter is trapped by thiobarbituric acid.

Widely used free radicals for assessing the effectiveness of antioxidants are hydroxyl radicals. These radicals are usually generated through the Fenton reaction (Scheme 4) using hydrogen peroxide. Then, the amount of hydroxyl radicals is detected via the reaction of thiobarbituric acid **7** and malondialdehyde **8** (Scheme 5), which leads to chromophore **9** (λ = 532 nm) [96]. The presence of antioxidants reduces the oxidation of 2-deoxy-d-ribose **10**. The principles of this method are well summarized by Apak et al. [97].

**Scheme 6:**
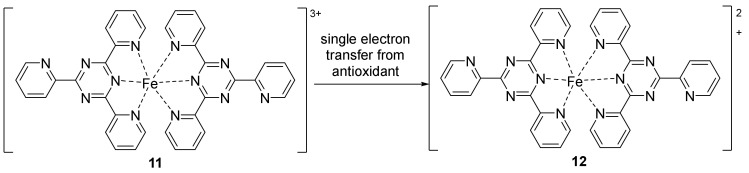
Reduction of Fe(III)–tripyridyltriazine complex **11** in the presence of antioxidants to deep blue-colored Fe(II)–tripyridyltriazine complex **12**.

The systems described above were elaborated based on hydrogen transfer (or sequential proton loss and electron transfer). Some tests involve electron transfer processes. A widely used method is the ferric-reducing antioxidant power (FRAP). This procedure involves antioxidant-induced reduction in Fe(III) to Fe(II), leading to blue-colored complex **12** (Scheme 6) with an absorption maximum of λ = 570 nm [98].

Another group of test methods are those for the evaluation of the antioxidant ability to complex with various metals. This property is of particular interest in the context of the Fenton reaction, which may occur in real systems. A typical assay involves the complexation of Fe(II) with antioxidants in the presence of ferrozine. When the analyte leads to a strong complex with Fe(II), no characteristic ferrozine complex with absorption at 562 nm is observed [99].

### 2.2. Determining Antioxidant Activity

Number of methods used for testing antioxidant activity in various lipid systems are available. Usually these methods are based on the analysis of the primary and secondary oxidation products. An overview of such methods is given by Abeyrathne et al. [100].

**Scheme 7:**
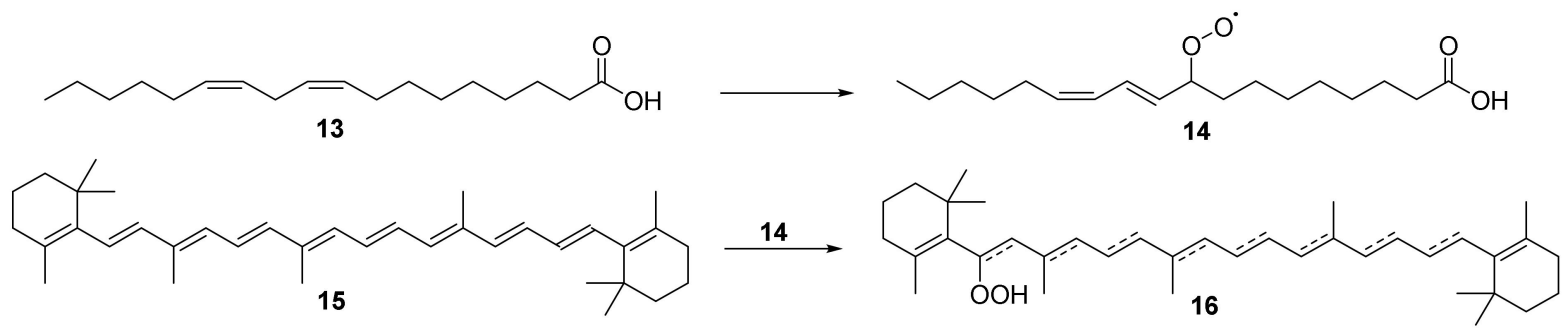
Linoleic acid **13** undergoes autooxidation, leading to linoleic acid peroxyl radical **14**. The latter induces the oxidation of *β*-carotene **15,** leading to colorless peroxide **16**.

One such method is the *β*-carotene bleaching test. Herein, the oxidation processes of linoleic acid **13** in the presence of air are studied, and the rate of degradation of carotene **15** at λ = 470 nm is monitored (Scheme 7) [101].

**Scheme 8:**
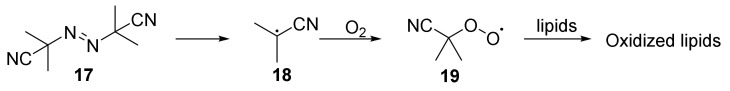
Generation of *C*-centered free radical **18** from AIBN **17** and sequential formation of peroxyl radical **19**.

In addition to air-induced methods, assays involving the controlled release of free radicals are elaborated. Such an example is the AIBN **17**-induced peroxidation of linoleates (Scheme 8) [102].

Similarly, antioxidants are tested not only in model systems but also in real samples. The substrates used for testing antioxidant efficiency are various vegetable oils and emulsions. The tests are run under different accelerated oxidation conditions. These methods are well summarized in some recent reviews [103,104,105].

## 3. Tautomerism of 1,3-Dicarbonyl Compounds

**Scheme 9:**
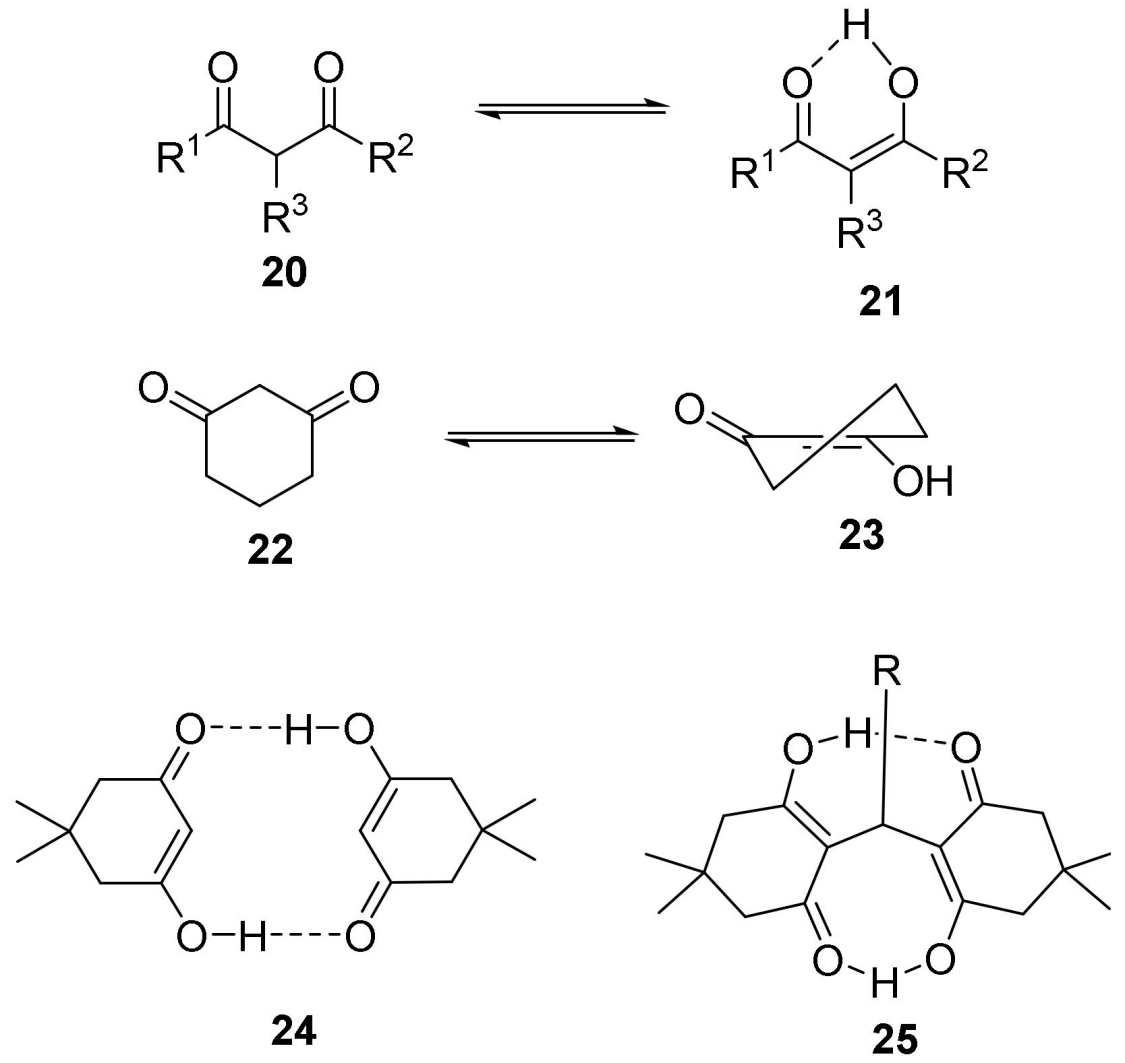
Keto–enol tautomerism: keto form of acyclic 1,3-dicarbonyl compound **20**, enol form of acyclic 1,3-dicarbonyl compound **21** representing intramolecular hydrogen bonds, cyclic 1,3-diketone **22**, twist form of cyclic 1,3-dicarbonyl compound **23**, intermolecular hydrogen bonds between two cyclic 1,3-diketones **24** and Vorländer adduct **25**.

It is well known that 1,3-dicarbonyl compounds exist in a tautomeric equilibrium between the keto- **20** and enol **21** forms (Scheme 9). Tautomerization is crucial from a medicinal chemistry viewpoint. The binding between small molecules with biological targets may strongly be affected by the keto–enol tautomerism of the compound [106]. Thus, understanding the keto–enol equilibrium might also be helpful in constructing antioxidants and/or free radical scavengers containing the 1,3-dicarbonyl moiety.

The scientific community has studied these equilibria in *β*-dicarbonyl compounds for more than a century—one of the first papers was published by Knorr in 1896 [107]. The general trends of the keto–enol equilibrium, both in 1,3-diketones and *β*-ketoesters, are well summarized by Iglesias [108]. The equilibrium between diketone and keto–enol forms is shifted by the temperature, solvents, various additives, and the structure of the 1,3-dicarbonyl compound.

In general, the enol form is facilitated due to the formation of both intra- and intermolecular hydrogen bonds. A cyclic six-member transition state, caused by an intramolecular hydrogen bond, favors the enol form in acyclic systems [109]. The formation of intramolecular hydrogen bonds is not sterically possible in cyclic 1,3-dicarbonyl compounds **22**. Such keto–enol tautomers exist as twisted structures **23** [110]. On the other hand, it is known that such enols in dimedone are stabilized by a network of intermolecular hydrogen bonds, leading to complex **24**. However, when two dimedone units are linked via a methylene bridge (so-called Vorländer adducts **25**), the structure is stabilized through intramolecular hydrogen bonds [111]. The tautomerization in cyclic 1,3-dicarbonyl compounds is affected by the size of the cycle—tautomerization does not occur in four-member cycles, while for other 1,3-dicarbonyl compounds, the activation energy is reduced by increasing the size of the cycle [112]. The enol form is dominant in diketones, while the keto form is characteristic of *β*-ketoesters. It has been observed that the introduction of an additional substituent in the *α*-position favors the enol form [108]. However, the diketone form is favored by *α*-fluorosubstituted diketones (compound **20**, R^3^ = F) [113]. Modifications of the substituents R^1^–R^3^ (structures **20** and **21**) in the diketone influence the ratio between the keto–enol and diketone forms; however, the former is still dominant [114]. The tautomeric equilibrium has also been studied for various curcumin derivatives: usually, the enol form is the dominant one; however, some exceptions are known. Both forms are present in similar amounts when an electron acceptor group is introduced to the benzene ring or a methyl group is introduced in the α-position [115].

Regarding the polarity of the media, the enol form in “inert” solvents is favored due lower polarity of the enol [116], whilst polar solvents facilitate the keto–tautomer [108]. Basic solvents (or additives of bases) for ketoesters shift the equilibrium to the keto tautomer, while for diketones—to the enol tautomer [116]. Usually, the addition of water mediates the tautomerization process [112].

The equilibrium of tautomeric forms is also affected by concentration [116]. An increase in temperature reduces the proportion of the enol form in favor of the diketone form [114]. The amount of keto form may be facilitated by UV irradiation [113], which may be significant for products exposed to light.

## 4. Acyclic 1,3-Dicarbonyl Compounds

### 4.1. Curcumin and Its Derivatives

Although curcumin demonstrates various beneficial properties, its application is limited due to its rather low stability. Its decrease in stability is attributed to two main aspects. One of them is the degradation caused by certain pH values, exposure to light, and temperature. These degradation products and general relationships are documented in a recent review article [117]. Briefly, the alkaline conditions, sunlight, and autooxidation lead to (a) scission of the C-C bond in 1,3-dicarbonyl moiety, leading to dehydrozingerone, ferulic acid, vanillin, and other low-molecular phenolic compounds, and (b) free radical-caused dimerization. Experimental data demonstrate that during storage, the amount of curcumin is reduced. However, no clear correlation between the degree of curcumin reduction and the antioxidant and/or antiradical activity is described.

In vivo applications of curcumin-containing compositions are subjected to enzymatic degradation, leading to different dehydrocurcumin derivatives [118,119]. The impact of a fully conjugated system on the antiradical activity of curcumin is analyzed here below. It should be noted that tetrahydrocurcumin, due to its lower autooxidation, exhibits better antioxidant properties in comparison to curcumin [119].

Due to the unique and wide biological activity of curcumin, it is essential to provide effective delivery systems that allow for transport through the gastrointestinal system and storing curcumin-containing products for their effective application. The latest trends in different delivery systems of curcumin are summarized by Chang et al. [120].

**Figure 2:**
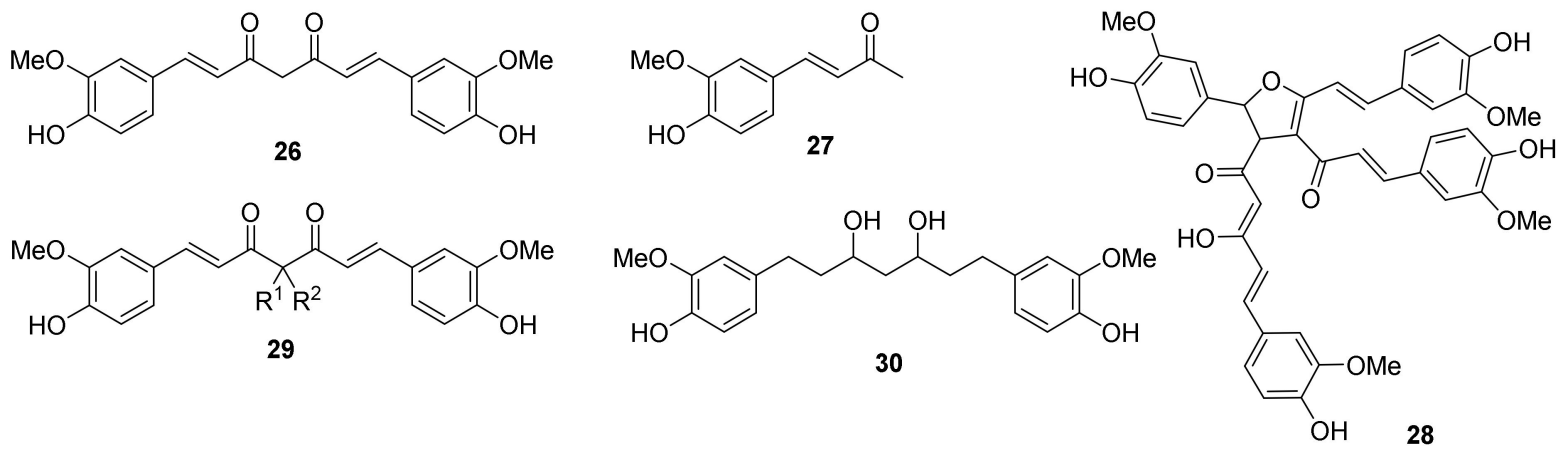
Structures of curcumin and its derivatives: curcumin (**26**), dehydrozingerone (**27**), radical dimerization product **28** of curcumin, α,α-disubstituted curcumin **29**, reduced form (“diol”) **30** of curcumin.

The antiradical activity of curcumin (**26**) is provided by both the aromatic rings (as a phenolic antioxidant) and the 1,3-carbonyl moiety (Figure 2).

For more than a decade, the reasons for providing curcumin’s antiradical and antioxidant activity have been discussed. Jovanovic et al. strongly support the role of the methylene moiety in reactions with free radicals. The reaction with the methyl radical justifies this: curcumin rapidly reacted with the radicals generated by pulse radiolysis, while dehydrozingerone **27** (half-curcumin) did not enter the reaction [121]. However, later studies with other radicals do not support this statement. Structure–activity studies of curcumin and its various derivatives have shown that hydroxy groups are crucial for donating a hydrogen atom to *N*- or *O*-centered free radicals. Curcumin inhibited 2,2′-azo*bis*(2-amidinopropane) (AAPH)-induced oxidation of deoxyribonucleic acid (DNA) and erythrocytes, and hemin-induced hemolysis of erythrocytes [122].

**Scheme 10:**
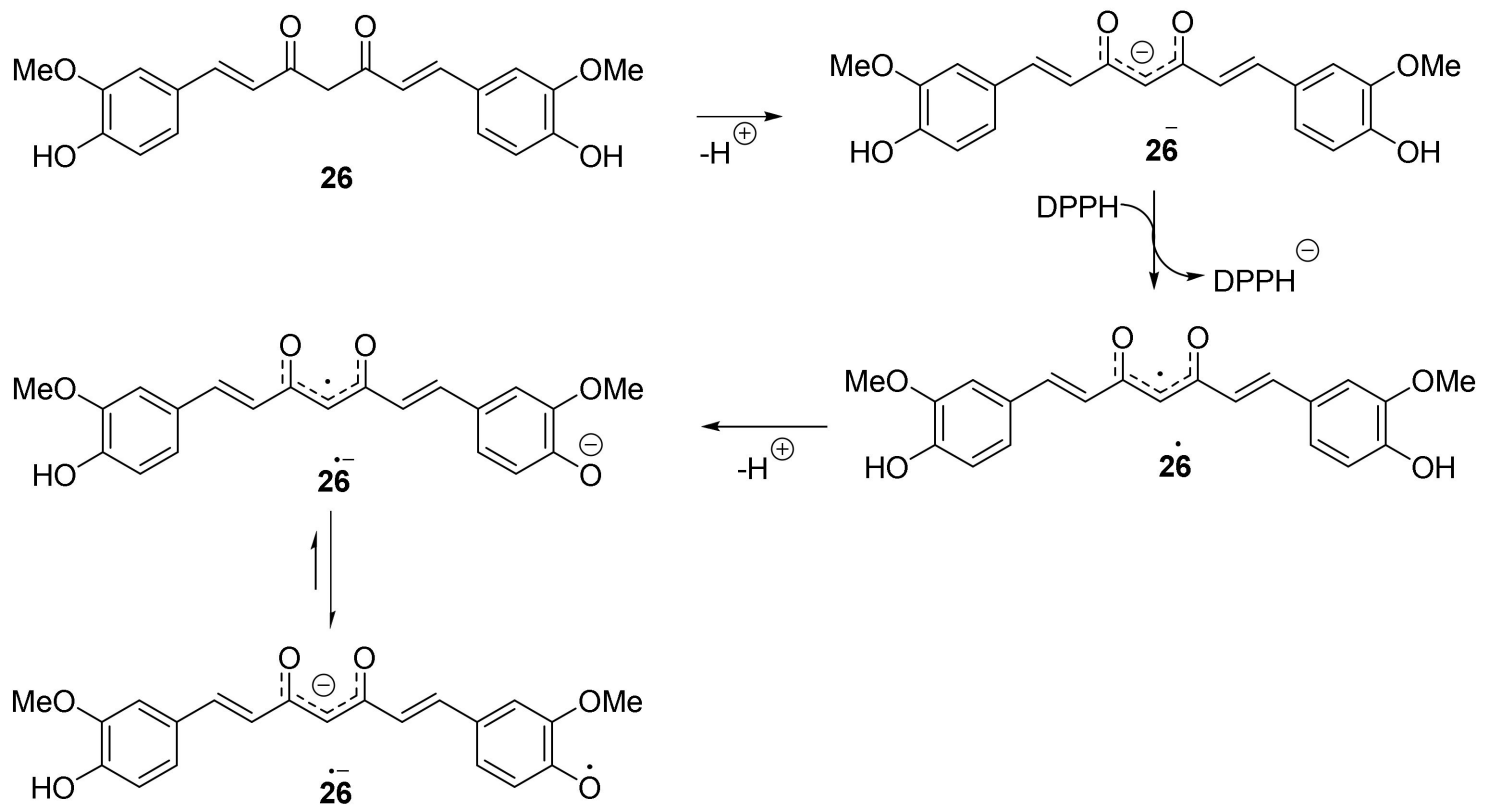
The plausible mechanism for the reaction between curcumin and DPPH. It is postulated that the first step is dissociation of curcumin **26** in the ions, leading to curcumin anion **26^−^**. Then, it is expected that an electron is transferred to the DPPH, leading to curcumin radical **26^•^**. The formed curcumin radical may undergo another proton loss, leading to either *O*- or *C*-centered radical anion **26^•−^**.

Meanwhile, the hydroxyl groups are not essential for reducing the 2,2′-azino-*bis*(3-ethylbenzothiazoline-6-sulfonic acid) (ABTS) cation radical: curcumin and *O*-benzyl-protected curcumin demonstrated similar behavior [122]. The azobisisobutyronitrile (AIBN)-initiated peroxidation of methyl linoleate and styrene was suppressed by curcumin, while derivatives without free hydroxyl groups did not reduce oxygen uptake. Thus, the unique role of hydroxyl groups over the enolate moiety for quenching peroxyl radicals was demonstrated [123]. Although a hard discussion on the radical formed from curcumin was published, the existence of a *C*-centered radical is firmly proved by the isolation of oxidation products. In addition to decomposition products (vanillin and ferulic acid), radical dimerization product **28** has been isolated [124]. Litwinienko and Ingold have well established the solvent effect on the reactivity of curcumin with 2,2-diphenyl-1-picrylhydrazyl (DPPH)—the reaction rate remarkably increased in solvents, which facilitated ionization of the antioxidant molecule, thus supporting the SPLET mechanism. These observations postulate that the first step is the formation of a mono-ion in the most acidic enol moiety, followed by electron transfer (Scheme 10) [125].

The role of the dicarbonyl moiety has been demonstrated via compounds **29** containing one or two methyl groups at the *α*-carbon. The monosubstituted compound **29** was 3- and 24-fold more reactive against DPPH than curcumin **26** and *α*,*α*-dimethyl curcumin. The increased reactivity of the monosubstituted derivative compared to curcumin might be due to the lower ionization potential of the former [126]. However, structure–activity correlations for curcumin derivatives give contradictory statements: according to the data provided by Somparn et al., not only the dicarbonyl moiety but also its reduced form (the corresponding diol **30**) may enhance the activity of these compounds in DPPH and linoleic acid oxidation assays [127]. Thus, the role of dicarbonyl fragments is diminished.

**Scheme 11:**
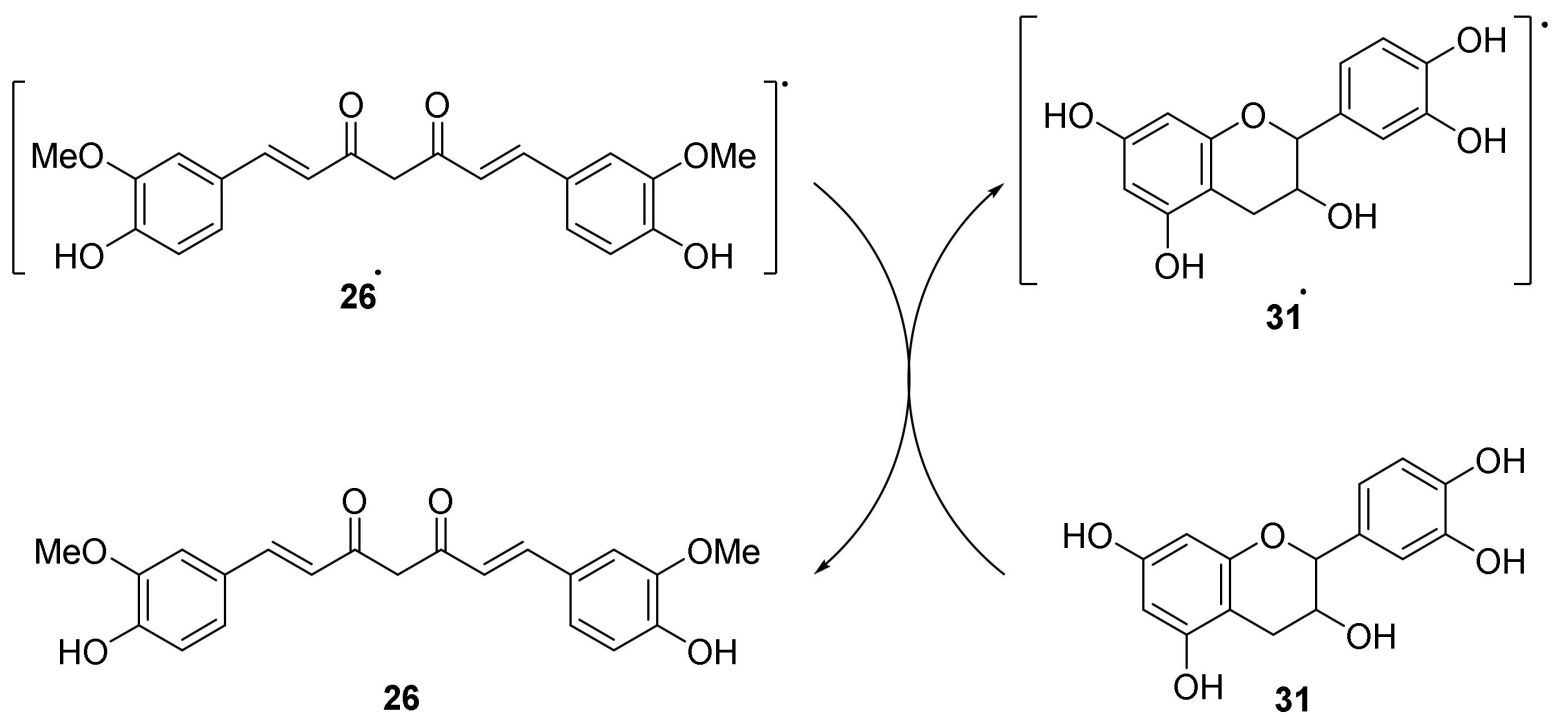
Plausible synergistic effect between curcumin **26** and (−)-epicatechin **31.** Curcumin **26** is postulated as the primary antioxidants, which quenches free radicals, leading to curcumin radical **26^•^**. Then, the curcumin may be regenerated through the reaction of curcumin radical **26^•^** and an antioxidant with a lower redox potential. Thus, (−)-epicatechin **31** regenerate curcumin **26** and the corresponding (−)-epicatechin radical **31^•^** by-product is formed.

Curcumin has demonstrated both antiradical activity and antioxidant activity in sunflower oil in various test systems. The oxidative stability of sunflower oil triglycerides increased by nearly 14-fold in the presence of a curcumin additive (1 mM) [128]. Curcumin effectively reduced the thermal and photo-induced oxidation of *β*-carotene in emulsions [129]. Aftab and Vieira demonstrated that curcumin is an effective antioxidant for the inhibition of heme-enhanced oxidation: curcumin was more than twice as active as resveratrol [130]. Curcumin has also been studied in various binary mixtures with other antioxidants. A weak synergistic effect (ABTS test) was observed for a mixture of curcumin **26** and (−)-epicatechin **31** or (−)-epicatechin-rich extract of green tea. The synergistic effect is explained by the regeneration of curcumin’s phenoxyl radical by an antioxidant with a lower redox potential (0.277 V and ~0.8 V for (−)-epicatechin and curcumin, respectively) (Scheme 11).

Binary antioxidant mixtures were tested to inhibit the autoxidation of sunflower oil. The results were unpredicted: the mixture containing (−)-epicatechin demonstrated a remarkable synergistic effect, while the mixture with the green tea extract even showed an antagonistic effect [128]. A green tea–curcumin drink helped lower the levels of serum redox, iron, and lipid peroxidation [131]. A synergistic effect was found for an equimolar mixture of curcumin and resveratrol in heme-enhanced oxidation of *N*,*N*,*N*′,*N*′-tetramethyl-1,4-phenylenediamine. This synergistic effect may be caused by the regeneration of curcumin by another antioxidant and the chelating of Fe(III) ions [130]. In addition to phenol-type antioxidants, ascorbate is also helpful in regenerating the parent molecule from the curcumin radical [132].

**Figure 3:**
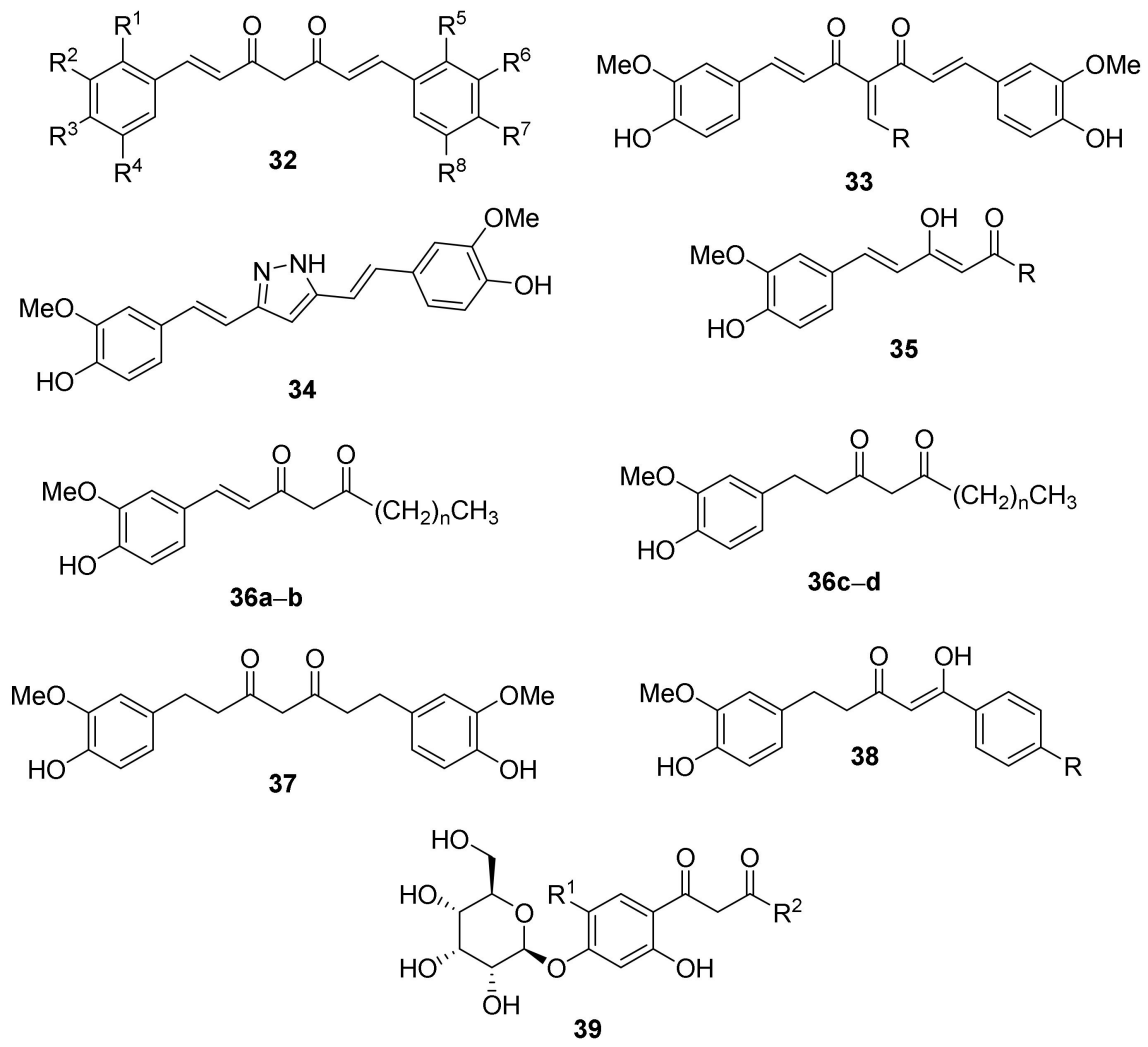
Synthetic analogs of curcumin: curcumin analogs **32** with various substituents in benzene rings, α-arylidene curcumin **33**, pyrazole moiety bearing curcumin derivative **34**, feruloyl acetone derivatives **35**, asymmetric derivatives of curcumin **36** found in ginger, tetrahydrocurcumin **37**, curcumin analogs with shortened conjugate chain **38**, *β*-d-glucopyranosyl modified acyclic 1,3-diketone derivatives **39**.

Hydroxy groups in the phenol rings significantly increase the antiradical activity of curcumin (Figure 3). Curcumin derivatives **32** with three or more hydroxy groups in the phenol rings (at least three of the substituent R^1^–R^8^ are hydroxy groups) show remarkable antiradical activity (inhibition concentration IC_50_ = 4.6–8.0 μM in the DPPH test) which is up to five times higher than for ascorbic acid (IC_50_ = 25.1 μM) and curcumin itself (IC_50_ = 21 μM). A hydroxy group in the *para*-position (in compound **32**, the R^3^ and/or R^7^ is OH) is crucial for high antiradical activity. In contrast, compounds with *ortho*-hydroxy-substituted (in compound **32**, the R^1^ and/or R^5^ are OH) and *para*-methoxy-substituted (in compound **32**, the R^3^ and/or R^7^ is OMe) aromatic rings do not significantly inhibit DPPH (IC_50_ > 500 μM). Electron-withdrawing groups, such as carboxylic acids, and their esters (in compound **32**, one of the substituents R^1^–R^8^ is COOH or COOAlk, IC_50_ > 100 μM) or a bromine atom (in compound **32**, one of the substituents R^1^–R^8^ is Br IC_50_ = 43 μM) tend to reduce the activity of the compound [133,134].

The presence of protons in the *α*-CH_2_ group is essential for the antiradical activity of 1,3-dicarbonyl compounds. Replacing these protons with an arylidene group significantly reduces the antiradical activity of curcumin—DPPH inhibition at a 100 μM concentration is 61% (in compound **33**, R = Ph) and 72% (in compound **33**, R = 4-HO-3-MeO-C_6_H_3_-). When replaced by a butylidene group (in compound **33**, R = C_3_H_7_), thus creating a less conjugated system than the arylidene substituent, the antiradical activity is even lower (43%). Curcumin inhibits 91% of DPPH at the same concentration [135]. It can be concluded that the overall degree of conjugation in the molecule is also crucial for the antiradical activity of curcumin.

Fully conjugated systems also play a vital role in ensuring antioxidant activity. For example, keto–enol tautomerization (thus creating a continuous conjugated chain between the phenol rings) is not possible in compound **34**. The antioxidant activity of this compound is higher when compared to curcumin, as evidenced by the electrochemical oxidation potential of these compounds (for curcumin, E^0^ = 0.66 V; for compound **34,** E^0^ = 0.62 V) [135].

Shortening of the conjugated system decreases antioxidant activity. For example, in compound **35**, where R = Ph or CF_3_, the IC_50_ is about two-fold lower than that of curcumin [134].

Asymmetrical derivatives of curcumin **36** are found in ginger. They show weaker antioxidant activity than curcumin in DPPH and lipid peroxidation tests. However, compared to the reference compounds, butylhydroxyanisole (BHA) and α-tocopherol, their activity against peroxides is relatively high. In this case, compounds with a higher degree of conjugation and a shorter alkyl chain are more effective. Metal ions in the solution influence the inhibition of peroxy radicals. In a test system where Fe^2+^ ions induce lipid peroxides, the antiradical activity of curcumin is much higher than for its asymmetrical analogs **36**. However, when using a test system without the presence of Fe^2+^ ions, this difference is much smaller. The activity of the most potent asymmetrical analog **36** is about 80% of the activity of curcumin. Considering that there are twice as many hydroxy groups in the aromatic system of curcumin, which may provide antiradical activity, the activity of compound **36** is very high. The lipophilicity of the alkyl chain can explain this in the ginger compound. It has also been observed that the activity of compound **36** decreases with the length of the alkyl chain [136].

Tetrahydrocurcumin **37** is a curcumin analog where conjugation is not possible. Still, it has also shown antioxidant activity: against certain types of radicals (e.g., peroxy and alkoxy radicals), it is even higher than that of curcumin (inhibition of lipid peroxidation at a 150 μM concentration for curcumin is 71%, for its derivative 18–83%). The hydrogenated form **37** has better bioavailability, and therefore, it is believed that a large part of dietary curcumin is converted and used in the form of tetrahydrocurcumin [137].

Less conjugated curcumin derivatives **38** (R is H, Me or *tert*-Bu) show weaker antioxidant properties than curcumin—the time to achieve the same concentration of lipid peroxidation products (peroxide value (PV) = 100 meq/kg) for compound **38** is two times shorter than that of curcumin. However, it has been observed that these compounds have a synergic effect with *α*-tocopherol. Furthermore, this effect is more significant than for the antioxidant mixture of ascorbic acid–tocopherol, which is currently used industrially. The induction period for lipid peroxidation for mixtures of compound **38** and tocopherol is more than 21 h, while for an ascorbic acid–tocopherol mixture at the same concentration, it is about 16 h. Other weak antioxidants could show similar properties in binary mixtures with more potent antioxidants [138].

Although many 1,3-dicarbonyl compounds have high antioxidant activity, they are mostly insoluble in water and have limited bioavailability. To solve this problem, *β*-d-glucopyranosyl derivatives **39** have been synthesized. Their antioxidant activity (DPPH radical inhibition of 74–85%) is weaker than that of ascorbic acid (98%), but the difference is not significant. Thus, water-soluble 1,3-dicarbonyl-type antioxidants with an activity similar to ascorbic acid could be obtained [139]. To increase the bioavailability, conjugates with pectin are also helpful. These macromolecules tend to self-assemble, leading to nanomicelles with improved stability compared to curcumin [140].

Various systems for delivering curcumin, in addition to the synthesis of water-soluble derivatives, have been developed to increase solubility. The bioavailability of curcumin was increased by introducing it to lecithin-based inverse hexagonal liquid crystals. The antiradical activity of such nanoformulations increased by about 100-fold and 10-fold compared to the free curcumin according to ABTS and DPPH tests, respectively [141]. The increased antiradical activity might be explained by lecithin and castor oil’s composition and minor ingredients. Chen et al. loaded curcumin onto CeO_2_/SiO_2_-PEG nanoparticles for its efficient delivery. Such systems showed antioxidant activity against H_2_O_2_-induced oxidative damage in Hep G2 cells [142]. Curcumin loaded onto mesoporous silica nanoparticles protected against ROS-induced cell damage [143]. Curcumin-loaded iron oxide nanoparticles reduced oxidative stress parameters in depressed rats [144]. A halloysite–cyclodextrin hybrid material was effective for the encapsulation of curcumin. The proposed material was influential in delivering curcumin and scavenging DPPH [145]. The loading of curcumin on both sodium caseinate (NaCas) and sodium caseinate–laponite (NaCas-LAP) nanoparticles leads to a more soluble form of curcumin. The carrier facilitated the antiradical activity of curcumin against ABTS 3–4-fold higher compared to curcumin. The results were more challenging in the DPPH test: the prepared nanoparticles demonstrated weaker antiradical activity than free curcumin, but a laponite additive to NaCas improved the free radical scavenging activity [146]. Curcumin has also been introduced in nano-emulsions, and its stability through the gastrointestinal tract has been established: it was observed that such emulsions preserve curcumin from degradation in the gastrointestinal tract, and their antiradical activity is greater than that of pure curcumin [147]. Lecithin-encapsulated curcumin demonstrated plasma antioxidant activity [148]. Another delivery system for curcumin with increased antiradical activity is a xanthan–starch matrix [149]. Chitosan-encapsulated nano-curcumin demonstrated in vivo reduction of arsenic-induced oxidative stress [150]. Curcumin loaded onto invitrogen cyanine3-labeled *N*-palmitoyl chitosan (Cy3-NPCS), and poly(1,4-phenyleneacetone dimethylene thioketal) (PPADT) nanoparticles effectively delivered medicine to the inflammation site and reduced oxidative stress [151].

**Figure 4:**
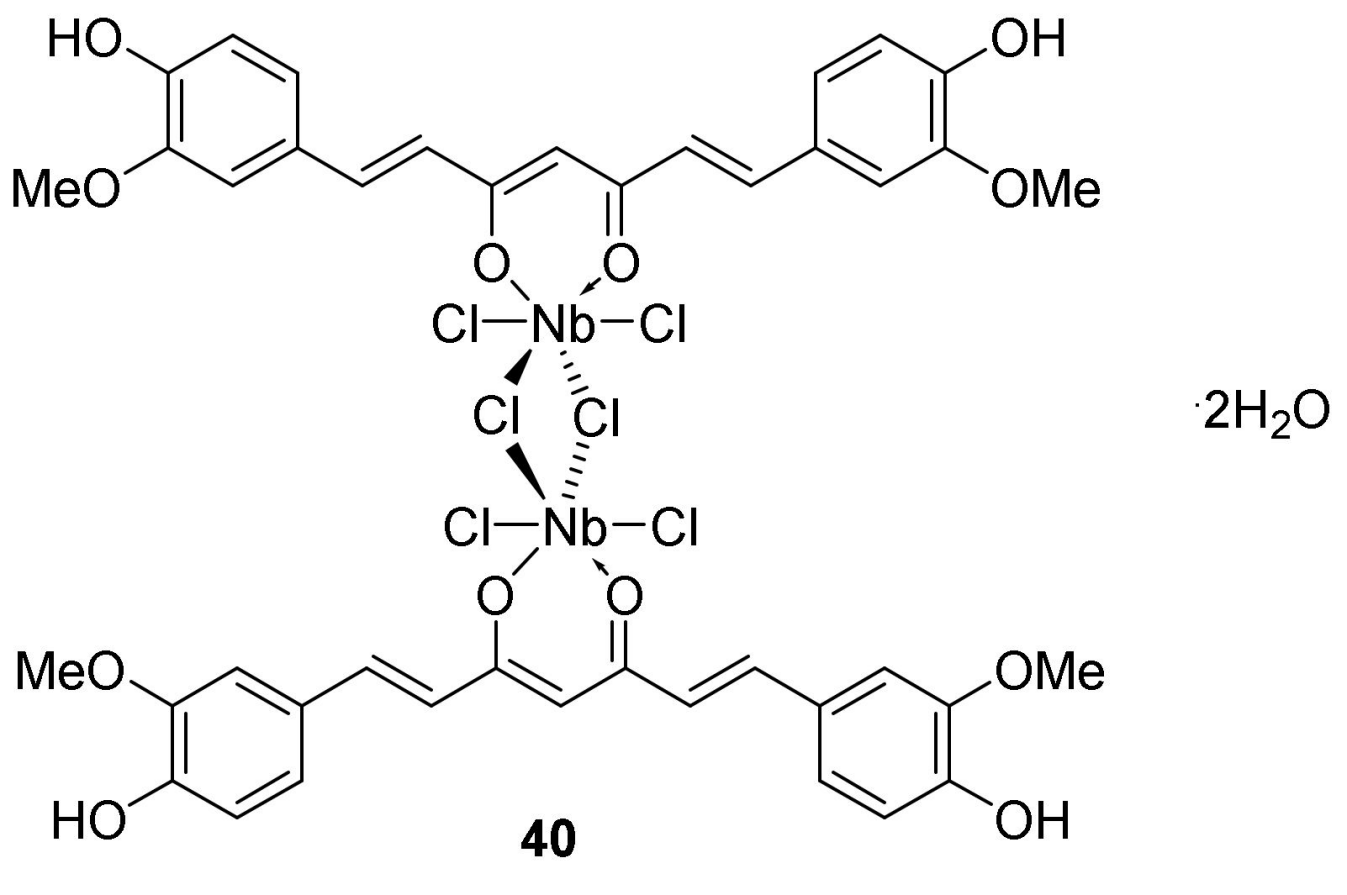
Niobium complex.

The antiradical activity of the curcumin niobium complex **40** (Figure 4) is comparable to that of ascorbic acid. In the DPPH test, the IC_50_ of the compound is 120 μg/mL (IC_50_ of ascorbic acid—101 μg/mL). Similar results are seen in the ABTS test, where the IC_50_ of the compound is 110 μg/mL (21% of the IC_50_ of ascorbic acid, when comparing molar concentrations) [152]. On the other hand, curcumin and calcium or magnesium complexes (two molecules of curcumin per cation) were synthesized. In these complexes, the counter ion did not influence the free radical scavenging activity (DPPH test) in comparison to curcumin [153].

### 4.2. Other Aliphatic 1,3-Dicarbonyl Compounds

**Figure 5:**
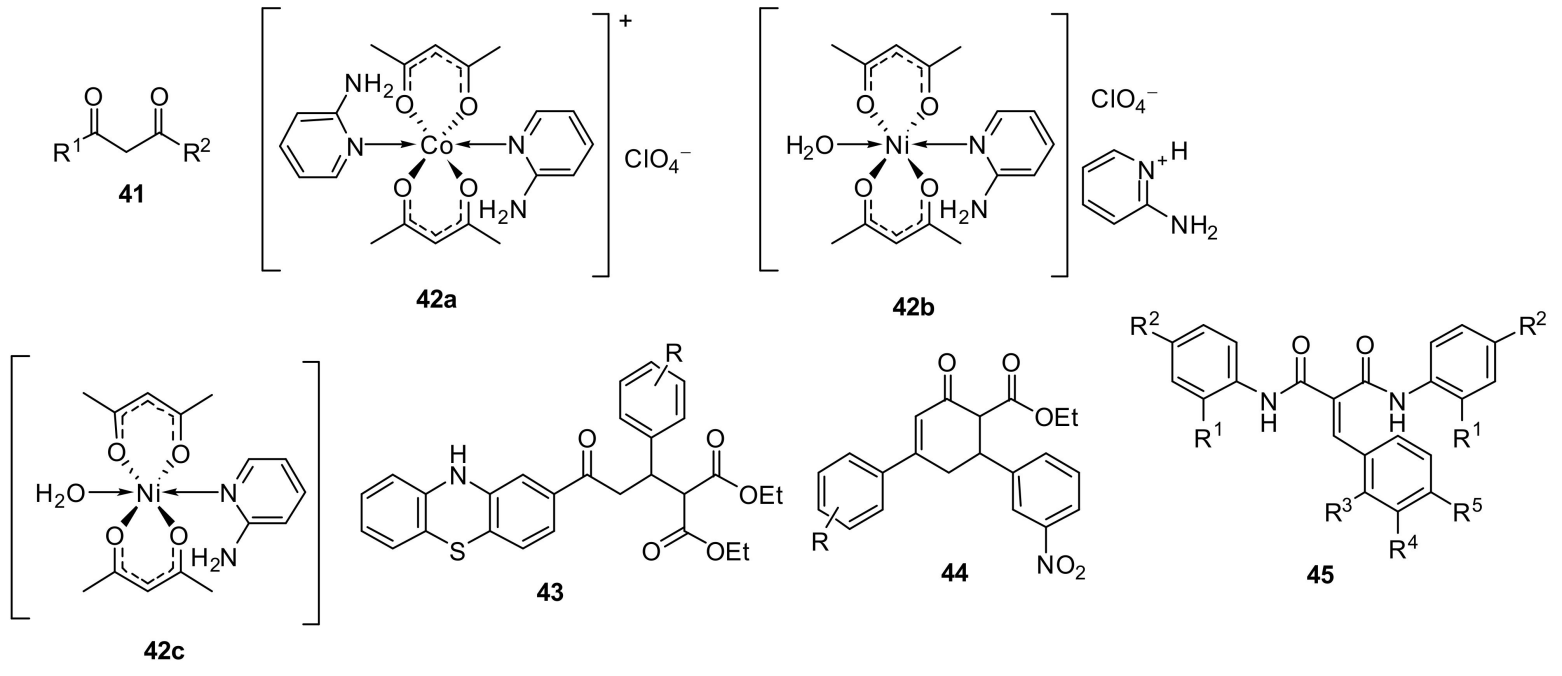
Other acyclic 1,3-dicarbonyl-type antioxidants: general structure of acyclic diketone **41**, acetylacetone complexes with transition metals **42**, *α*-monosubstituted malonate derivative **43**, curcumin–malonate hybrid **44** and arylidene malonamides **45**.

Eucalyptus leaves contain various aliphatic 1,3-diketones **41** (Figure 5). These compounds do not have a phenol group, so their antioxidant activity is provided solely by the 1,3-diketone moiety. The most active of them is n-tritriacontane-16,18-dione (R^1^ = R^2^ = C_15_H_31_), which shows higher antioxidant properties against peroxy radicals than both butylated hydroxytoluene (BHT) and tocopherol in water/ethanol systems. However, it did not show any inhibition of autooxidation in oil systems. This means that the presence of water is essential for the antioxidant activity of these compounds and keto–enol tautomerization may be essential, wherein the enol form mainly provides the activity. Tautomerization is only one of the factors determining the antioxidant activity of a compound. The alkyl chain length is essential too; e.g., the simplest *β*-diketone acetylacetone (compound **41** R^1^ = R^2^ = CH_3_) has the same keto–enol form ratio as for n-trithriacontan-16,18-dione (1:6), but this compound shows antioxidant properties neither in the oil nor the water/ethanol system [154]. Both R^1^ and R^2^ in diketone **41** should be long alkyl chains to ensure antioxidant activity. This is demonstrated by the asymmetric compound **41** (R^1^ = CH_3_ and R^2^ = C_11_H_23_), which, similarly to acetylacetone, does not show antioxidant properties.

A *β*-diketone, which contains an additional hydroxy group (in the compound **41** R^1^ = C_15_H_31_, R^2^ = (CH_2_)_11_CH(OH)CH_2_CH_2_CH_3_) shows similar activity to its analog without a hydroxy group. This means that hydroxy groups (and the presence of an “isopropanol” unit—a scaffold that is well-known due to its high affinity to oxygen) in the alkyl chains do not affect activity but may potentially improve water solubility, thus making the compound applicable in various water–oil mixtures, such as foods [155].

In the ferric ion-reducing antioxidant power (FRAP) test, both Co(III) and Ni(II) complexes with acetylacetone have higher antiradical activity than ascorbic acid. The anions of the complex play a role in ensuring the overall antioxidant activity of the resulting chelates. For example, the antioxidant activity of complex **42c** is about twice as high as that of complex **42b**. The Co(III) complex **42a** has a lower activity than the Ni(II) complex with the same ligand, which means that the nature of the metal also influences the activity—oxidation state, size, and other factors [156].

Malonate derivative **43** contains a benzyl group at the α-carbon. The benzyl group’s type and position of substituents affect the antiradical properties. The unsubstituted compound **43** (R = H) has good antioxidant activity in the ABTS test (IC_50_ = 40.0 μg/mL), which is comparable to the well-known natural antioxidant quercetin (IC_50_ = 44.5 μg/mL). Antiradical activity increases if an electron-donating moiety is present in the benzene ring in the *para*-position (e.g., compound **43** R = 4-Me, IC_50_ = 36.0 μg/mL). If there is an electron-withdrawing group in the *para*-position (e.g., compound **43** R = 4-CHO, IC_50_ = 43.0 μg/mL), it decreases. If the electron-withdrawing group is in the *meta*-position (e.g., compound **43** R = 3-NO_2_, IC_50_ = 41.2 μg/mL), the antiradical activity is similar to the unsubstituted analog [157].

Compounds **44** could be considered curcumin and malonate hybrids. However, the antiradical activity of these compounds is fragile (in the DPPH test, the IC_50_ is 4–10-fold higher than that of ascorbic acid). The reason could be a steric hindrance and/or electronic effects, but the exact reason is unknown [158].

The antiradical activity of other malonate derivatives **45** is weak: DPPH radical inhibition at a 1:1 molar ratio (100 μM concentration) is around 0.5–7.0%. The only case where slightly better activity was achieved was when R^4^ = MeO and R^5^ = OH (inhibition of 51%, IC_50_ = 110 μM). However, the compound most likely acts as a phenol-type antioxidant in this case. Like the curcumin compounds, the lack of *α*-protons appears to suppress compounds **26** from acting as 1,3-dicarbonyl-type antioxidants [159].

## 5. Cyclic 1,3-Dicarbonyl Compounds

### 5.1. Carbocyclic 1,3-Dicarbonyl Compounds

**Figure 6:**
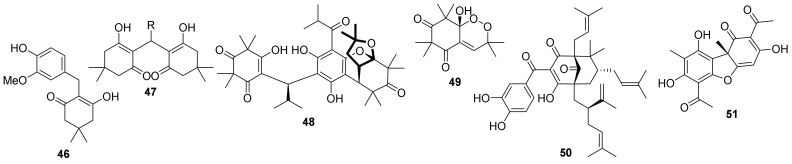
Various cyclic 1,3-diketone-type antioxidants: arylmethyl substituted dimedone **46**, *bis*-dimedone derivatives **47**, myrtucommuacetalone **48**, G3-factor **49**, garcinol **50,** and usnic acid **51**.

One of the simplest carbocyclic 1,3-dicarbonyl compounds is 1,3-cyclohexadione (Figure 6). Arylmethyl dimedone **46** shows good antiradical activity against DPPH (IC_50_ = 23.0 μM), which is comparable to ascorbic acid (IC_50_ = 25.7 μM) and *tert*-butylhydroquinone (IC_50_ = 19.5 μM). In the galvinoxyl test, its activity was even greater when compared to the reference compounds: the IC_50_ for compound **46** was 20.3 μM, which is comparable only to *tert*-butylhydroquinone (IC_50_ = 22.6 μM). The activity of other commonly used antioxidants, such as ascorbic acid (IC_50_ = 83.0 μM) or *α*-tocopherol (IC_50_ = 39.2 μM), under the same conditions, was considerably lower [160].

Additionally, compounds containing two 1,3-diketone moieties were elaborated. Tetraketone **47** shows antioxidant properties. However, no clear relationship has been observed between the position of the substituents in the benzene ring (R = Ar) and the antiradical activity of the compounds. For example, in the DPPH test, one of the most active compounds was *meta*-bromophenyl-substituted tetraketone (IC_50_ = 52.4 μM), but an analog containing chlorine showed no antiradical activity. A cinnamic aldehyde derivative where R is a styryl group exhibited the highest antioxidant activity (IC_50_ = 33.6 μM), which is higher than that of the reference antioxidant *tert*-butyl-4-hydroxyanisole (IC_50_ = 44.7 μM). Compound **47**, where R is BrC = CHPh, also has relatively high activity (IC_50_ = 58.4 μM), so an extended conjugated system may positively affect the antiradical activity of the compound [161].

The activity of tetraketone derivative **47,** where R is a 3,5-dimethylphenyl group, is comparable to ascorbic acid. In the DPPH test, the *bis*-dimedone derivative inhibited 82% of free radicals (concentration of 100 μg/mL), while ascorbic acid inhibited 80%. When expressed in molar concentrations, approximately twice as much ascorbic acid as *bis*-dimedone is needed to inhibit an equal amount of DPPH radicals [162].

Several compounds containing a 1,3-dicarbonyl group have been isolated from *Myrtus communis* plants. Compounds **48** and **49** have demonstrated a good inhibition capacity against NO∙radicals. Compound **49** inhibits 59% NO∙ at a 25 μg/mL concentration, which is comparable to the reference compound, NG-methyl-l-arginine acetate, which inhibits 66%. Compound **48** is an even more effective inhibitor (inhibiting 82% of NO∙) [163].

Some compounds contain several 1,3-dicarbonyl units. One such antioxidant is garcinol. Similarly to compounds **48** and **49**, garcinol **50** showed moderate antioxidant activity in the DPPH test (approximately 85% of the inhibition of ascorbic acid). However, its NO∙radical scavenging activity was more significant than for ascorbic acid. It is also a somewhat effective superoxide anion scavenger compared to gallic acid [164,165].

Usnic acid **51** has shown moderate antioxidant activity. In the DPPH test, its IC_50_ value (49.5 μg/mL) was more than five times that of *α*-tocopherol (IC_50_ = 9.8 μg/mL). However, the O_2_^−^ scavenging ability of usnic acid and *α*-tocopherol is comparable (IC_50_ = 20.4 and 21.0 μg/mL, respectively) [166]. It has been reported that the interaction of usnic acid with a human blood cell culture promotes mild cell apoptosis and ROS levels and stimulates DNA synthesis. The activity of ursnic acid strongly depends on the concentration; at higher concentrations, it shows cytotoxicity, oxidative stress, and other harmful effects [167].

**Figure 7:**
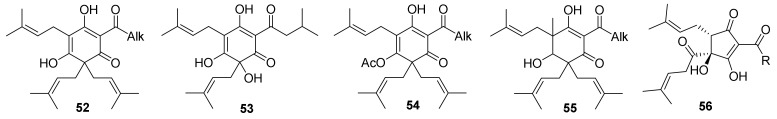
Hop acids: lupulones **52**, humulones **53**, acylated lupulones **54**, methyl-substituted lupulones **55**, and iso-*α*-acids **56**.

Carbocycles containing 1,3-dicarbonyl moieties are also found in hops (Figure 7). The antiradical activity (DPPH test) of lupulones **52** and humulones **53** is similar to that exhibited by α-tocopherol and ascorbic acid. It is worth mentioning that acylation of the hydroxy group and the introduction of an additional methyl group (compounds **54** and **55**, respectively) increases the activity of the compounds by up to 10 times. These compounds effectively reduced rat brain lipid peroxidation [168]. The presence of bitter acids is essential for providing antioxidant and/or antiradical activity in hop extracts. The inhibition of hydrogen peroxide increases with the amount of humulone in the extract. More humulone is a more effective scavenger than the phenol-type antioxidant rutin [169]. Wietstock et al. analyzed the antioxidant behavior of bitter acids during wort boiling. A separate analysis of various ingredients showed similar free radical trapping for both *α*- and *β*-acids, while iso-*α*-acid **56**—a heat-induced isomerization product of α-acid—even showed some prooxidant effects. The free radical-scavenging activity of the α-acids was attributed to the presence of three *β*-keto units, which facilitated the formation of stabilized phenoxyl radicals [170]. In addition, the Fenton and the Haber–Weiss reactions may be limited by the complexation of Fe(II) and Cu(I) ions [171]. Due to the complexation of iron ions, *α*- and iso-*α*-acids protected 2-deoxyribose from oxidative degradation. It should be highlighted that *α*-acids were more effective [170]. Karabin et al. indirectly reported on the antioxidant activity of iso-*α*-acids. The analysis of iso-*α*-acids during the storage of lager beer showed a reduction in both the compounds and the antioxidative activity [172]. In contrast, other research indicated that beers with a lower amount of iso-*α*-acids demonstrate higher antioxidant activity according to an oxygen radical absorbance capacity (ORAC) test [173]. Humulone- and lupulone-rich hop extract demonstrated antioxidant activity against reactive oxygen species in cells under simulated solar irradiation [174]. *β*-Acids have increased free radical-scavenging properties of chitosan films: a 0.1–0.3% additive of *β*-acids to the film increases the inhibition of DPPH to 55–75%. Furthermore, these films have been found to be valuable covers for soybean oil packages, thus reducing the autoxidation processes in the oil (the peroxide value of the non-filmed oil after 25 days was >25 meq O_2_/kg, while the peroxide value of the samples covered with *β*-acid-improved chitosan film was around 10 meq O_2_/kg) [175].

### 5.2. O-Heterocyclic 1,3-Dicarbonyl Compounds

#### 5.2.1. Dihydropyran-2,4-Diones

**Figure 8:**
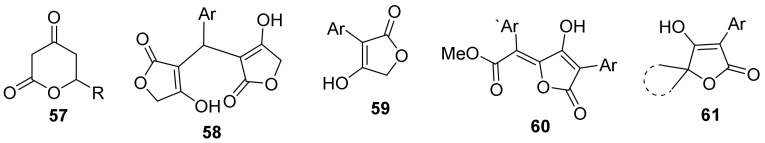
Antioxidants with dihydropyran-2,4-diones moieties.

Overall, the DPPH radical scavenging activity of compound **57** is relatively weak (Figure 8). However, it can be observed that in cases where R = *para*-MeOC_6_H_4_, the activity of this compound was even weaker. This means that the substituent in the fifth position of the dihydropyran ring affects the activity of the compound [176].

5-Membered lactones **58**–**61** have been described, too. In silico and in vitro DPPH assays have turned pulvinic acid derivatives into promising antioxidants. A slight increase in antiradical activity was observed when several moieties of tetronic acid **58** were introduced into the structure [177]. Compounds with the general structure **60** demonstrated good thymidine and plasmid DNA protection against reactive oxygen species and Fenton-type oxidative systems [178].

#### 5.2.2. Meldrum’s Acid Derivatives

**Figure 9:**
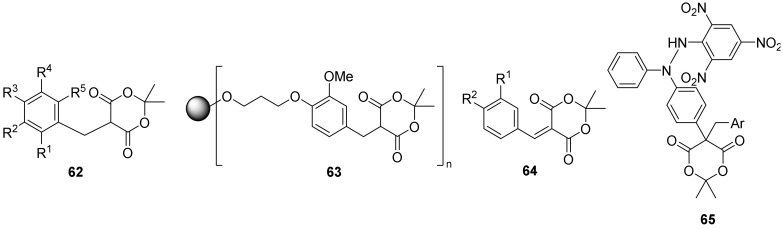
Antioxidants with Meldrum’s acid residue: arylmethyl Meldrum’s acids **62**, dendrimers decorated with arylmethyl Meldrum’s acid units **63**, arylidene Meldrum’s acids **64**, and arylmethyl Meldrum’s acid–DPPH conjugate **65**.

Various arylmethyl Meldrum’s acids have shown good antioxidant properties (Figure 9). It has been observed that a hydroxy group at the *para*-position of the aryl group (in compound **62**, R^3^ = OH) improves the activity of the compound (e.g., for the compound, where R^3^ = OH and R^2^ = OMe, IC_50_ = 20.3 μM, but if R^3^ = OH and R^2^ = R^4^ = Ome, then IC_50_ = 14.5 μM). A fluorine atom (compound **62**, R^3^ = F, IC_50_ = 18.2 μM) and an acetoxy group (compound **62**, R^3^ = OAc, IC_50_ = 16.8 μM) in this position gives a similar effect. Conversely, a methoxy (compound **62**, R^3^ = OMe, IC_50_ = 26.7 μM) or nitro (compound **62**, R^3^ = NO_2_, IC_50_ = 35.0 μM) *para*-substituted arylmethyl Meldrum’s acids have relatively low activity [179]. Some dendritic architectures **63** containing arylmethyl Meldrum’s acid moieties as surface groups have also been studied. Among them, the derivatives with a flexible glycerol core were determined to be a promising scaffold for elaborating powerful antioxidants [180]. The role of *C*-centered radicals was proven via trapping the radicals with DPPH with di-substituted Meldrum’s acid **65** [179].

In contrast, arylidene Meldrum’s acids **64**, compared to their reduced analogs **62**, have low antioxidant activity. However, by varying the type and position of the substituents in the aromatic ring, it is possible to improve the activity, e.g., in compound **64**, where R^1^ = OMe and R^2^= CH_2_CH_2_CH_2_Br, IC_50_ = 55.6 μg/mL in the DPPH test. However, it was still significantly less than the reference compound—ascorbic acid (IC_50_ = 6.4 μg/mL) [181]. Compounds with a hydroxy group in the *para*-position and a methoxy (compound **64**, R^2^ = OH and R^1^ = OMe, IC_50_ = 24 μg/mL) or ethoxy group (compound **64**, R^2^ = OH and R^1^ = OEt, IC_50_ = 39 μg/mL) in the *meta*-position showed good antiradical activity in the DPPH test in comparison to ascorbic acid (IC_50_ = 22 μg/mL). In these cases, activity is most likely due to the phenol fragment, and the compound acts as a phenol-type antioxidant [182].

#### 5.2.3. Coumarin and Its Derivatives

**Scheme 12:**
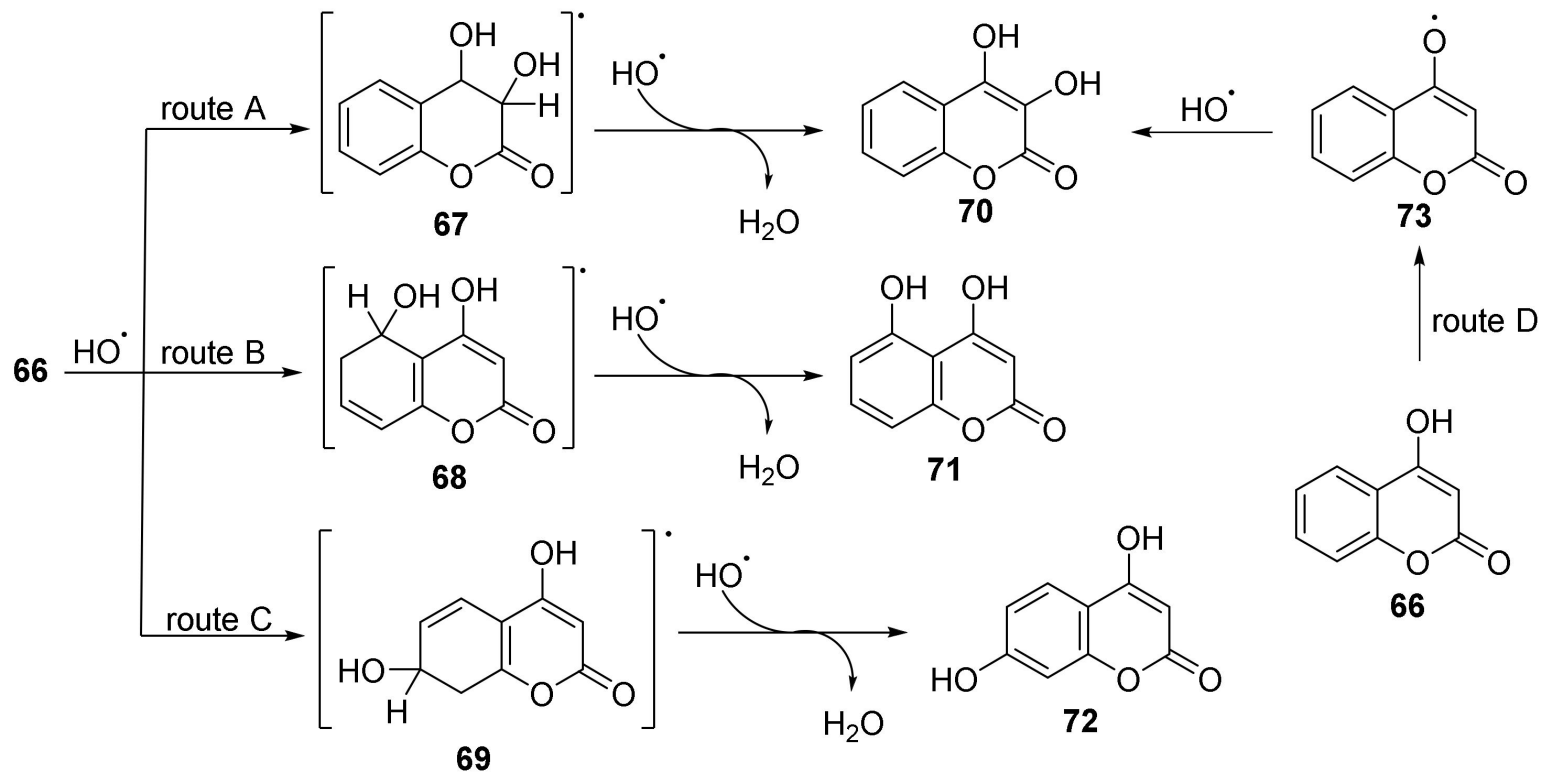
The plausible mechanism for the reaction between coumarin **66** and hydroxyl radical. Several pathways for the reaction between 4-hydroxycoumarin and hydroxyl radical are provided. Route A foresees the reaction of the hydroxyl radical with the 1,3-dicarbonyl moiety, followed by abstraction of the hydrogen atom, leading to 3-hydroxyl coumarin **70**. The same compound can be obtained through route D: the first step would be homolytic dissociation of the OH bond and a following reaction with hydroxyl radical. Routes B and C foresee the addition of hydroxyl radical to the benzene ring, leading to intermediates **68** and **69**, and sequential re-aromatization of the system leads to 5- or 7-hydroxy coumarins **71** and **72**.

**Figure 10:**
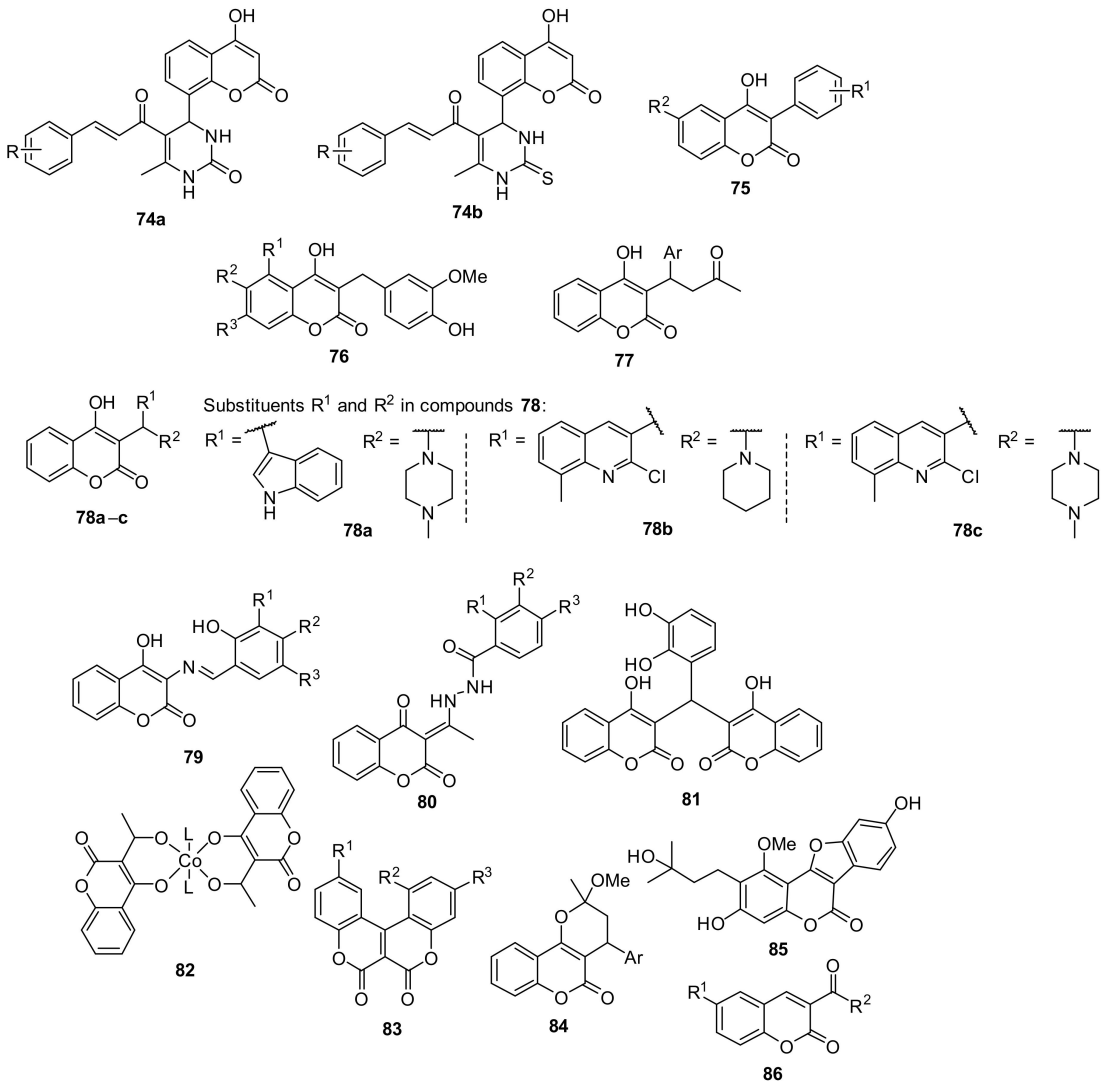
4-Hydroxycoumarin derivatives: 8-substituted 4-hydroxycoumarins **74**, 3-aryl-4-hydoxycoumarins **75**, 3-arylmethyl-4-hydroxycoumarins **76** and **77**, amino group containing 3-hetarylmethyl 4-hydroxycoumarins **78**, 3-benzylideneamino-4-hydroxycoumarins **79**, 3-aminomethylene-4-hydroxycoumarins **80**, *bis*-(4-hydroxycoumarins) **81**, 3-acetyl-4-hydroxycoumarin–Co complex **82**, coumarin derivatives with rigid **83** or masked **84** and **85** 1,3-dicarbonyl units, and 3-acylcoumarin **86**.

Unsubstituted 4-hydroxycoumarin **66** (Figure 10) has moderate antiradical activity in the DPPH test (EC_50_ = 3.8 mM) [183,184,185]. Unsubstituted coumarin also effectively scavenges HO^•^. Electron paramagnetic resonance (EPR) spectra analysis has demonstrated the presence of carbon-centered radicals. It has been suggested that 4-hydroxycoumarin reacts with HO^•^ through radical adduct formation, followed by hydrogen atom abstraction. Compound **70** can be formed through sequential hydrogen atom transfer and radical-radical coupling (Scheme 12) [185].

8-Substituted compounds **74** containing a 4-dihydropyrimidin-2(1*H*)-one or 4-dihydropyrimidin-2(1*H*)-thione unit can be considered 3-unsubstituted 4-hydroxy coumarins. The activity of these compounds varied from 9 to 78% (DPPH test), although a strong correlation between the substituents and the antiradical activity was not observed [186].

The activity of 3-phenyl-4-hydroxycoumarin is slightly lower (EC_50_ = 4.2 mM) than for the unsubstituted 4-hydroxycoumarin. Compounds **75,** where R^2^ = H and R^1^ = electron-withdrawing group (EWG), showed similar activity to 3-phenyl-4-hydroxycoumarin, for example, chlorine-substituted compounds (in compound **75,** R^1^ = *ortho*-Cl, EC_50_ = 4.14 mM or R^1^ = *para*-Cl, EC_50_ = 4.15 mM). The antioxidant activity of a naphthyl-substituted 4-hydroxycoumarin **75** (4-hydroxy-3-naphtyl coumarin) was even weaker.

If there is an electron-donating group (EDG) in the benzene ring at the *meta*- or *para*-position (compound **75**, R^1^ = *ortho*- or *para*-EDG), the antiradical activity increases [183,184]. The results of the ORAC test show that a *para*-methyl group (compound **75**, R^1^ = *para*-CH_3_, ORAC = 6.5) or a *meta*-hydroxy group (compound **75**, R^1^ = *meta*-OH, ORAC = 4.9) containing 3-phenyl-4-hydroxycoumarins have a higher antioxidant activity than the unsubstituted 4-hydroxycoumarin (**66**, ORAC = 4.2).

Among compounds **75**, 6-chloro-4-hydroxy-3-(3-hydroxyphenyl) coumarin (R^1^ = 3-OH, R^2^ = Cl) shows the highest activity. The activity of this compound (ORAC = 7.7) is comparable to the reference compound quercetin (ORAC = 7.3). The presence of an EWG group in the coumarin aromatic ring (compound **75** R^2^ = Cl) also improves the antioxidant activity of 4-hydroxycoumarin [187]. The activity of compounds may be affected not only by the electronic characteristics of the substituents but also by steric hindrance. Smaller molecules with better steric availability to the DPPH radical tend to have higher antiradical activity [183].

3-Arylmethyl substituted coumarins **76** also possess considerable antioxidant activity. The vanillin derivatives **76** are more active (IC_50_ = 10.0–36.4 μM) against DPPH radicals than their parent molecules **66** (inhibition at a 1:1 ratio with DPPH is below 15%). Furthermore, the 6-methyl substituted compound **76** (R^2^ = Me, IC_50_ = 36.4 μM) shows weaker antioxidant properties than its unsubstituted analog (R^2^ = H, IC_50_ = 10.0 μM). The activity of 6-unsubstituted arylmethyl coumarin **76** is even greater than for such antioxidants as ascorbic acid (IC_50_ = 25.7 μM) and *tert*-butylhydroquinone (IC_50_ = 19.5 μM) [183]. It appears to be a tendency for electron-withdrawing groups in the coumarin aromatic ring to increase the antioxidant activity of coumarin derivatives [187], while EWGs decrease it [188]. Several 3-benzyl 4-hydroxy coumarins **77** were analyzed in silico. The results correlate with the experimental data—they are promising structures for constructing powerful antioxidants. According to the preliminary theoretical calculation, the activity does not strongly depend on the substituents [189].

The substitution of 4-hydroxycoumarin in the third position with various nitrogen-containing heterocycles through a methylene group produces compounds with good antioxidant properties. The antiradical activity of compounds **78a**–**c** in the DPPH test (IC_50_ = 10.5–11 μM) is higher than that of ascorbic acid (IC_50_ = 12.5 μM). Perhaps in these compounds, the methyl groups provide additional radical stabilization in 4-methylpiperazine and the eighth position of quinoline. Other compounds of this type also show good antioxidant properties. Most of them possess higher activity than BHT [190].

The antiradical activity of 4-hydroxycoumarin **79** with an imine moiety in the third position is generally weaker than curcumin (IC_50_ = 39.6 μM). Only in some cases, e.g., compounds **79**, where R^1^ = OH (IC_50_ = 48.5 μM), R^1^ = R^2^ = OH (IC_50_ = 45.8 μM), and especially, R^3^ = NO_2_ (IC_50_ = 38.6 μM), the IC_50_ value is comparable to the reference compound. This shows that the position of the OH groups in the benzene ring is important. The presence of a nitro group may also positively affect the antioxidant activity of the compound. Conversely, replacing OH groups with OMe, Me groups, or halogens reduces the activity of compounds **79** [191]. Antonijevic et al. have studied some similar compounds **80**, containing hydrazide residue. Compounds that could be considered the derivatives of vanillic acid (R^1^ = H, R^2^ = OMe, R^3^ = OH) demonstrated superior DPPH inhibition compared to other tested compounds. Thus, the role of phenol-type antioxidants in these structures was highlighted [192].

Dicoumarol **81** shows weak antioxidant activity. After 20 min inhibition of ABTS was only 40% at a 2 mM concentration. However, the intramolecular hydrogen bonds within the molecule may hold it in a suitable position to bind to enzymes in biological systems, helping them interact with other antioxidants [193].

4-Hydroxycoumarin chelates **82** show excellent antioxidant properties as well. For compounds **82** (L = H_2_O or L = OEt), the IC_50_ in the DPPH test was 0.022 μmol/L and 0.021 μmol/L, respectively, while for the 4-hydroxycoumarin ligand itself, IC_50_ = 0.059 μmol/L [194].

Wang et al. also studied coumarin-fused coumarins **83**. Although the compounds could be considered 1,3-dicarbonyl compounds, they act as phenol-type antioxidants. Theoretical calculations have revealed compound **83** (R^1^–R^3^ = OH) as the most active one—the compound may be involved even in two HAT or SPLET steps. The compounds in nonpolar media react via the HAT mechanism, but in polar, the SPLET mechanism is preferable [195]. Theoretical calculations for a set of compounds **84** containing a fused cycle, thus restricting keto–enol tautomerism in the 4-hydroxycoumarin system, have been determined to be promising agents for DPPH inhibition [189]. The plausible explanation for the activity of these compounds might be the presence of a benzylic position. A hidden enolate moiety can be seen in glycyrurol **85** isolated from *Glycyrrhiza uralensis*. This coumarin derivative effectively reduced MPP^+^-induced reactive oxygen species in PC12D cells. However, it did not demonstrate any significant effect in the *β*-carotene bleaching test and the DPPH system [196].

Few compounds **86** containing a 3-carboxy group, and thus bearing an exocyclic fragment of the 1,3-dicarbonyl compound, have been studied. However, their antiradical activity against DPPH was provided due to 6-hydroxy groups (the inhibition of DPPH reached 60%) [197]. Similar systems were studied by Vazquez-Rodriguez et al. The hydroxyl radical scavenging activity of these compounds is comparable to well-known antioxidants, like quercetin and catechin. However, the activity of these compounds should be attributed to the free hydroxyl groups [198].

### 5.3. N-Heterocyclic 1,3-Dicarbonyl Compounds

**Figure 11:**
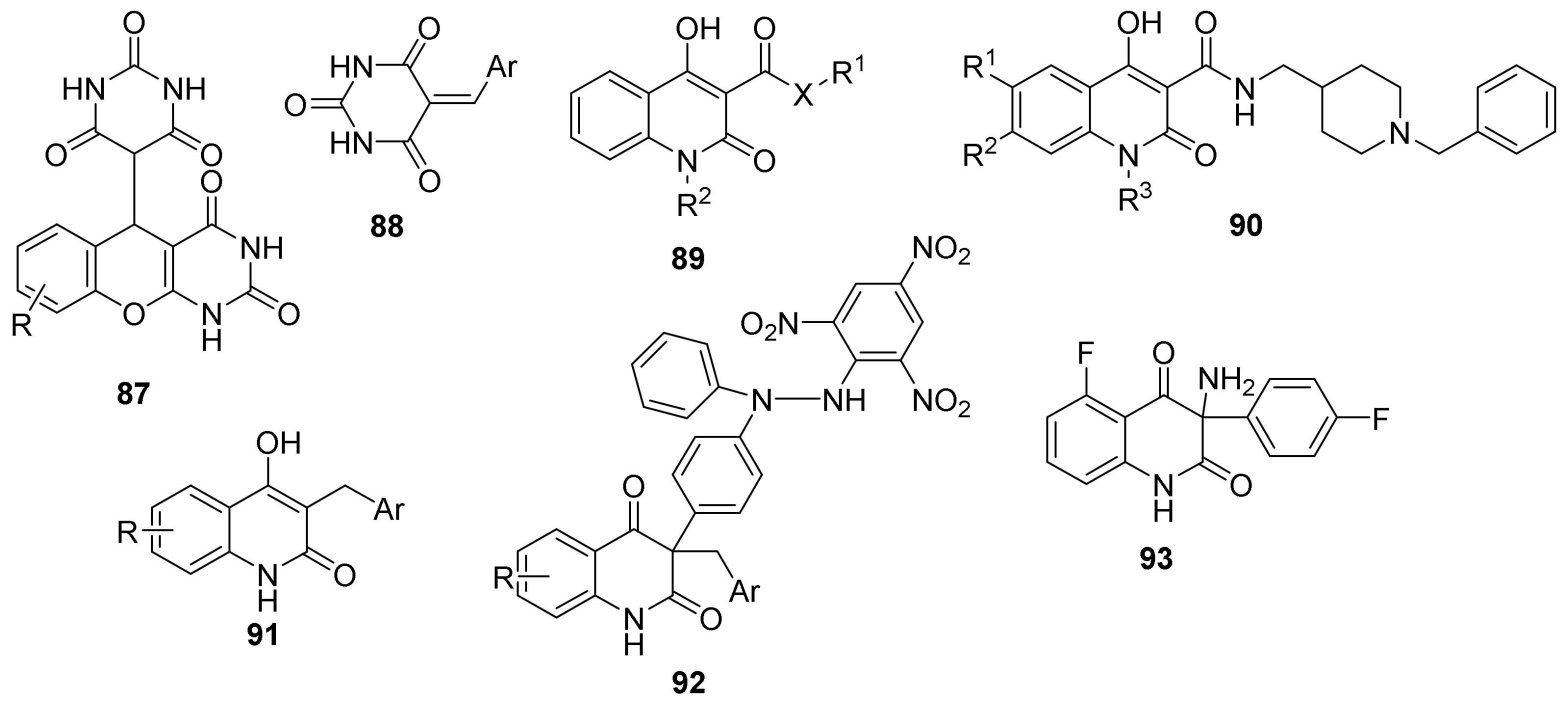
1,3-Dicarbonyl-type antioxidants with *N*-heterocycle moieties: derivatives of barbituric acid **87** and **88**, 3-acyl-4-hydroxyquinolin-2-ones **89** and **90**, 3-arylmethyl-4-hydroxyquinolin-2-one **91**, 3-arylmethyl-4-hydroxyquinolin-2-one and DPPH conjugate **92,** and 3,3-disubstituted quinolin-2,4-dione **93**.

Herein, two groups of *N*-heterocycles are studied (Figure 11): derivatives of barbituric acid and 4-hydroxyquinolinones. The non-substituted compound **87** (R = H) possesses antiradical activity against DPPH (76% inhibition at a 100 μg/mL concentration), which is comparable to ascorbic acid (inhibition is 80%). The presence of halogens in the benzene ring (in compound **87** R = Hal) reduces activity, while a methoxy group (in compound **68** R = OMe) improves activity [162]. In silico and in vitro data have revealed arylidene barbiturates **88** to be promising free radical scavengers [177].

DPPH test results have shown that compounds **89,** where X = NH and R^1^ = 4-NH_2_C_6_H_4_, have high antiradical activity. After 20 min at a concentration of 0.1 mM, the DPPH inhibition of these compounds (R^2^ = C_6_H_5_ or CH_3_) was 90%, which was higher than for the reference compound nordihydroguaiaretic acid (NDGA) (81%). These compounds have the position of amine in the benzene ring in common. Compared to other compounds of this type, the increased activity is ensured by the absence of intramolecular forces between the primary amine and the amide carbonyl group. Although the antiradical activity in the DPPH test of other derivatives **89** is negligible, compounds where X = O, R^1^ = CH_3_, R^2^ = C_6_H_5_ or X = NH, R^1^ = 2-NH_2_C_6_H_4_, R^2^ = C_6_H_5_ showed excellent results in a HO^•^ quenching test. Their inhibitions were 95% and 98%, respectively. For comparison, Trolox inhibited 88% under the same conditions. The previously mentioned *para*-amino phenyl compounds **89** showed weak activity in the OH^•^ test (21% and 10%) [199].

The antiradical activity of quinoline derivatives **90** can be improved by introducing hydroxy groups into positions 6- and 7- of the quinoline ring. Compound **90** without free hydroxy groups (R^1^ = R^2^ = OMe and R^3^ = H) did not show antiradical activity in the DPPH test, while the activity of compound **90** (R^1^ = R^2^ = OH and R^3^ = H, effective concentration, EC_50_ = 12.2 μM) is comparable to that of quercetin (EC_50_ = 10.3 μM). Compounds **90** with R^3^ = Me (EC_50_ = 21.7 μM) or R^3^ = Bn (EC_50_ = 20.8 μM) also showed high activity (higher than curcumin, EC_50_ = 26.6 μM), but the activity of these compounds was weaker than that of compound **90**, where R^3^ = H. The *N*-substituent in the quinoline ring also affects the activity of compound **90**: *N*-unsubstituted quinoline is a better antioxidant than the *N*-methyl- or *N*-benzyl substituted analogs **90** [200].

3-Arylmethyl 4-hydroxyquinoliones **91** are effective DPPH and GO quenchers. Although their activity strongly depends on the substituents in the arylmethyl moiety, the presence of 1,3-dicarbonyl moiety is reasonable—the corresponding product **92** was isolated [201].

Antioxidants that perform a specific function are also important—inhibiting certain radicals from treating specific medical disorders. For example, when *cis*-platinum is used in cancer treatment, patients often experience hearing impairment caused by additional ROS generation. Compound **93** has shown an ability to inhibit these ROS, thus preventing hearing damage [202].

## 6. Conclusions

The 1,3-dicarbonyl unit is found in many compounds with antiradical and antioxidant properties. Evidence for the crucial role of the 1,3-dicarbonyl moiety for its antiradical activity has been demonstrated for various compounds in different test systems. On the other hand, competing studies are suggesting the dominant role of the substituents in the benzene ring, thus revealing these compounds as antioxidants—mainly as classical phenol-type antioxidants. However, the current information has revealed 1,3-dicarbonyl compounds as promising scaffolds for promising antioxidants. Further investigations into this series of compounds should result in excellent antioxidants due to the presence of acidic C-H bonds, thus leading to compounds that may be easily adjusted both for lipophilic and polar media. The general correlation between the structural elements and antiradical/antioxidant activity are as follows: (a) both acyclic and cyclic α-monosubstituted 1,3-dicarbonyl compounds demonstrate increased activity in comparison both with unsubstituted and *α*,*α*-disubstituted compounds; (b) the acyclic structures are more sensitive to various structural modifications; (c) the antiradical activity of cyclic 1,3-dicarbonyl compounds are less affected by the structure of the heterocycle. Thus, cyclic 1,3-dicarbonyl compounds could be more effective for constructing powerful antioxidants.

## Data Availability

Not applicable.
